# Endophytic *Pseudomonas pergaminensis* DR1 controls anthracnose and promotes growth of medicinal orchid *Pleione bulbocodioides*

**DOI:** 10.3389/fmicb.2026.1909299

**Published:** 2026-10-12

**Authors:** Yun-Qiao Xie, Kai-Feng Tao, Neng-Qi Li, Jian Yue, Juan Su, Jiang-Yun Gao

**Affiliations:** 1 State Key Laboratory of Vegetation Structure, Functions and Construction (VegLab), Institute of Biodiversity, School of Ecology and Environmental Science, Yunnan University, Kunming, China; 2 Institute of International Rivers and Eco-Security, Yunnan University, Kunming, China; 3 Guangnan Yaowanggu Biological Technology Company Limited, Wenshan, China

**Keywords:** anthracnose, biocontrol, *Colletotrichum orchidophilum*, plant growth-promoting traits, *Pleione bulbocodioides*, *Pseudomonas pergaminensis*

## Abstract

*Pleione* species are valued ornamental plants and the source of the traditional Chinese medicine “Bingqiuzi.” In this study, pathogenic fungi were isolated from diseased leaves and pseudobulbs of *Pleione bulbocodioides* (Franch.) Rolfe in Yunnan Province, China. Through Koch’s postulates, two pathogenic strains were obtained and identified as *Colletotrichum orchidophilum* based on morphological characterization and multilocus phylogenetic analyses (*ITS*, *CHS*, *TUB2*, *GAPDH*, and *ACT*). Notably, the two strains exhibited significant differences in sporulation capacity and conidial length. An antagonistic bacterial strain, DR1, was isolated from the roots of wild *P. bulbocodioides* and identified as *Pseudomonas pergaminensis* based on phylogenetic analysis of 16S rRNA and housekeeping genes (*gyrB*, *rpoB*, and *rpoD*). Strain DR1 exhibited strong antagonistic activity against *C. orchidophilum* in dual culture assays, with an inhibition rate of up to 71.28%, and demonstrated the ability to produce siderophores and proteases. The volatile organic compounds (VOCs) produced by DR1 achieved inhibition rates of 53.04% and 48.08% against the two pathogenic strains, respectively. In greenhouse trials, 4 months after application of the DR1 suspension, the disease index was significantly reduced, with a control efficacy of 62.5% (beta regression model), and the number of newly emerged leaves increased by 198% compared with the control (*P* < 0.01). Based on the genomic information of the *P. pergaminensis* type strain, the potential mechanisms by which DR1 may promote plant growth and suppress the pathogen were preliminarily discussed. This study conducted a preliminary identification of *C. orchidophilum* causing anthracnose on *P. bulbocodioides* and demonstrated that *P. pergaminensis* DR1 has promising biocontrol and growth-promoting potential, providing a valuable microbial resource for the sustainable cultivation of medicinal orchids.

## 1 Introduction

The genus *Pleione* comprises herbaceous orchids characterized by pseudobulbs that store water and nutrients. It includes approximately 33 species and nine natural hybrids, predominantly distributed in China (Wu et al., 2019). The dried pseudobulbs of *P. bulbocodioides* and *P. yunnanensis* constitute the botanical source of the traditional Chinese medicine “Bingqiuzi,” which is recognized for its anti-asthmatic and anti-inflammatory effects (Zhou et al., 2025; Cao et al., 2025; Ji et al., 2024). Recent studies have also identified antitumor phenanthrenes and bibenzyls in this genus (Liu et al., 2008; Wu et al., 2019). Driven by its values, the commercial demand for *Pleione* has increased steadily in recent years. However, like most orchids, *Pleione* produces minute seeds that lack sufficient reserves, resulting in low natural germination rates (Nogueira et al., 2025). Overharvesting, habitat destruction, and low reproductive rates have led to severe population declines (Liu et al., 2015; Babbar and Singh, 2016). Artificial propagation primarily relies on slow pseudobulb division (1–3 new bulbs per plant per year) or *in vitro* culture; however, plantlets derived from tissue culture are weak and susceptible to diseases, particularly as cultivation areas expand (Ji et al., 2024; Hsu et al., 2024).

*Colletotrichum* species rank among the world’s top ten fungal pathogens, causing leaf spots, stem blights, and fruit rots in a wide range of hosts (Chang et al., 2023; Zhang et al., 2023). Several *Colletotrichum* species have been reported on orchids, with anthracnose emerging as a significant threat to *Pleione* cultivation. For example, Chai et al. (2023) identified *C. fructicola* as the causal agent infecting *P. formosana*. However, the causal agent of a recent severe anthracnose outbreak on *P. bulbocodioides* in Yunnan Province has yet to be characterized.

Endophytic bacteria-microbes that colonize internal plant tissues without causing disease-can promote plant growth and enhance stress resistance through direct mechanisms (e.g., nitrogen fixation, phosphate solubilization, siderophore production, phytohormone synthesis) and indirect mechanisms (e.g., antimicrobial compounds, hydrolytic enzymes, and induction of systemic resistance) (Sharma et al., 2025; Tarigan et al., 2023; Dobrzyński and Jakubowska, 2025; Saikia and Thakur, 2024). In orchids, *Pseudomonas* and *Bacillus* species are frequently isolated and have been shown to exhibit biocontrol activity against *Colletotrichum* and *Fusarium* pathogens (Lalanne-Tisné et al., 2023; Saikia et al., 2022). Moreover, orchid endophytic bacteria can promote the growth of non-orchid crops (Tedsree et al., 2022) and improve the acclimatization efficiency of orchid seedlings (Cristiano et al., 2023). Recently, Dong et al. (2026) demonstrated that *C. orchidophilum* is the causal agent of anthracnose on *P. yunnanensis*, and their *in vitro* assays showed that the biocontrol fungus *Trichoderma virilente* Z53 can inhibit this pathogen by up to 80.25%. Although the study primarily focused on genomic and metabolomic analyses and did not include field trials on *P. bulbocodioides* to evaluate disease control efficacy, it represents an advance in understanding the pathogen and its biocontrol. Nevertheless, no studies have yet reported the isolation of biocontrol bacteria from *Pleione* or systematically evaluated their efficacy against *Pleione* anthracnose.

To address the knowledge gaps outlined above, this study aimed to: (1) isolate and identify the causal pathogen; (2) screen for antagonistic bacteria from wild *P. bulbocodioides* roots; and (3) evaluate their biocontrol efficacy and plant-growth-promoting potential through both *in vitro* and greenhouse assays. These findings provide both a theoretical foundation and a valuable microbial resource for the sustainable management of *P. bulbocodioides* anthracnose.

## 2 Materials and methods

### 2.1 Sample sources

Diseased *P. bulbocodioides* samples were collected from a plantation in Qujing City, Yunnan Province (25.615°N, 103.826°E). In 2025, a disease outbreak occurred in the plantation, with an incidence of 20%–30%. After surveying the entire affected area, visual estimation indicated that more than 80% of the diseased plants exhibited a typical symptom complex, including chlorotic leaves with yellow halos, dark brown lesions along the veins (occasionally forming concentric rings), and shriveled, brittle leaf tissues. Severely affected plants also showed sunken brown lesions with black dots on the pseudobulbs. Based on these observations, a single plant displaying the above typical symptoms was collected and transported to the laboratory for pathogen isolation and identification.

Samples of healthy wild *P. bulbocodioides* roots, rhizosphere soil (within 0.5 cm of the roots), and leaves were collected from the Alpine Botanical Garden in Lijiang City, Yunnan Province (27.217°N, 100.181°E) for the isolation of biocontrol bacteria. Five samples of each tissue or soil type (roots, rhizosphere soil, and leaves) were collected. After sampling, the soil was carefully backfilled to preserve the integrity of the wild *P. bulbocodioides* pseudobulbs, thereby minimizing disturbance to the wild population.

### 2.2 Isolation and identification of the pathogen from *P. bulbocodioides*

#### 2.2.1 Pathogen isolation and purification

The pathogen was isolated using the tissue isolation method. Diseased samples were rinsed with sterile water, air-dried, and surface-disinfected with 75% ethanol. Under a laminar flow hood, tissue pieces of approximately 0.5 cm × 0.5 cm were excised, sequentially disinfected with 75% ethanol for 30 s and 1% sodium hypochlorite solution for 5 min, and then rinsed three times with sterile water. Excess moisture was removed with sterile filter paper, and the tissue pieces were placed onto PDA medium (potato 200 g, glucose 20 g, agar 15 g, distilled water 1000 mL) plates and incubated at 28 °C for 2-4 days. Emerging hyphae from the margins were picked and purified.

#### 2.2.2 Pathogenicity test (Koch’s postulates)

Pathogenicity was verified according to Koch’s postulates. Healthy living plants were used for inoculation. Pseudobulbs and leaves were surface-cleaned, disinfected with 75% ethanol, and wounded with a sterile pin (approximately 10 punctures). Fungal plugs (5 mm in diameter) taken from 7-day-old purified cultures on PDA plates were placed mycelium-side down onto the wounds, while sterile PDA agar plugs served as controls. Strains isolated from pseudobulbs were individually inoculated onto three pseudobulbs, and strains isolated from leaves were individually inoculated onto three leaves. The inoculated pseudobulbs and leaves, with the plants kept intact, were planted in seedling trays filled with sterilized substrate, covered with matching tray lids to retain moisture for 2 days, and then maintained at room temperature for 10 days. Once symptoms resembling those of the original diseased samples developed on the inoculated plants, the pathogens were re-isolated from symptomatic pseudobulbs and leaves. The re-isolated strains were purified and identified by internal transcribed spacer (*ITS*) sequencing, as described in Section “2.2.4 DNA extraction, PCR amplification, and sequencing”; pairwise sequence alignments using NCBI BLAST (blastn) were performed between the *ITS* sequences of the re-isolated strains and those of the respective original inoculation strains to verify their identity.

#### 2.2.3 Morphological observation

The morphological identification of the pathogen was performed by culturing on potato dextrose agar (PDA) at 28 °C under a 12-h light/12-h dark photoperiod. Colony diameters were measured on days 3, 5, 8, and 10 post-inoculation using the cross-streak method, with three replicate plates per strain per time point, and the experiment was repeated three times. For sporulation assays, 5-mm mycelial plugs were excised from the colony margins and transferred onto fresh PDA plates. On days 3, 5, 8, 10, 13, and 15 of incubation, each plate was flooded with 20 mL of sterile distilled water; mycelia were scraped off using a sterile pipette tip, and the suspension was thoroughly mixed to obtain the spore suspension. 10 μL of the spore suspension were loaded onto a hemocytometer and counted under a light microscope. The results from five independent experiments served as the replicates (n = 5). For spore size measurement, five colonies cultured for 20 days were used for each strain. Spore photographs were taken under a light microscope, and 30 randomly selected spores per plate were measured for length and width using ImageJ software; the length-to-width ratio of each spores was then calculated, giving a total of 150 spores measured per strain.

#### 2.2.4 DNA extraction, PCR amplification, and sequencing

For molecular identification, genomic DNA was extracted from the original isolates that had been confirmed pathogenic by Koch’s postulates and from the re-isolated strains obtained after inoculation, using a fungal DNA extraction kit (Solarbio, Beijing). PCR amplification of the *ITS* region was performed with the universal primers ITS1 and ITS4 (White et al., 1990). The PCR amplification was carried out using 2× Accurate Taq Master Mix (dye plus) (Accurate Biotechnology, Hunan, China, Cat. No. AG11010). Each reaction mixture contained 6.5 μL of 2× Accurate Taq Master Mix (dye plus), 0.5 μL each of forward and reverse primers (10 μM), 1 μL of DNA template, and nuclease-free water to a final volume of 13 μL. The thermal cycling conditions followed the corresponding reference (White et al., 1990). The PCR products were examined on 1% agarose gels and sequenced by Sangon Biotech (Shanghai, China). Sequences were assembled using SeqMan software and subjected to BLAST searches in the NCBI database. To further determine the taxonomic status of the pathogenic isolates that had fulfilled Koch’s postulates, multi-gene sequencing was performed on the original isolates, including chitin synthase (*CHS*), beta-tubulin (*TUB2*), glyceraldehyde-3-phosphate dehydrogenase (*GAPDH*), and actin (*ACT*) (Matos et al., 2025; primer sequences are listed in Supplementary Table 1). The PCR system for amplification of the *CHS*, *TUB2, GAPDH*, and *ACT* genes was the same as described above, and the thermal cycling conditions followed Matos et al. (2025).

#### 2.2.5 Phylogenetic analysis

All phylogenetic analyses were performed in PhyloSuite v1.2.3 (Zhang et al., 2020). Sequences of each gene locus were aligned with MAFFT (Katoh and Standley, 2013), and the resulting alignments were trimmed by trimAl (Capella-Gutiérrez et al., 2009). Trimmed multiple-gene alignments were concatenated via the Concatenate Sequence module. The maximum-likelihood (ML) phylogenetic tree was reconstructed using the built-in IQ-TREE module (Nguyen et al., 2015) under an Edge-linked partition scheme (Chernomor et al., 2016). The substitution-model option was set to Auto, and ModelFinder (Kalyaanamoorthy et al., 2017) was automatically invoked to select the best-fit nucleotide-substitution model for each partition based on the AIC criterion. Branch support was assessed by 1000 ultrafast bootstrap replicates (UFBoot2; Hoang et al., 2018).

#### 2.2.6 *In vitro* toxicity assay of chemical fungicides against *C. orchidophilum*

The inhibitory activity of six fungicides against *C. orchidophilum* strains 2-PT5 and 2-PT6 was determined using the mycelial growth rate method. The fungicides were selected as follows: five fungicides with reported efficacy against *Colletotrichum* sp. were chosen based on Xie (2024), including 25% pyraclostrobin suspension concentrate (SC), 250 g/L difenoconazole emulsifiable concentrate (EC), 450 g/L prochloraz emulsion, oil in water (EW), 25 g/L fludioxonil SC, and 90% carbendazim wettable powder (WP); in addition, a copper-based fungicide, 100 g/L copper sulfate aqueous solution (AS), was included. Detailed information on all fungicides and the assay concentrations is provided in Supplementary Table 3. Briefly, fungicide-amended PDA plates were prepared at a gradient of concentrations, with sterile water serving as a blank control. A 5-mm mycelial plug from an actively growing colony was placed in the center of each plate, with three replicates per concentration. Plates were incubated at 25 °C until the colony diameter in the blank control reached approximately three-quarters of the plate diameter. Colony diameters were measured by the cross method (Yang et al., 2017), and inhibition rates were calculated by Equation 1. Toxicity regression equations were established using DPS software (v9.5), with the logarithm of fungicide concentration as the independent variable (x) and the probit of inhibition rate as the dependent variable (y). From these, EC_50_, EC_95_ values, and their 95% confidence intervals were calculated.

### 2.3 Isolation, screening and identification of biocontrol bacteria

#### 2.3.1 Isolation and screening of biocontrol bacteria

Roots and leaves of wild *P. bulbocodioides* were processed separately for endophyte isolation. After rinsing with sterile water and surface disinfection with 75% ethanol, the tissues were cut into small pieces, placed in a sterile mortar, and thoroughly ground with 5 mL of sterile water to prepare a tissue homogenate. One milliliter of the homogenate was serially diluted to 10^−6^. Aliquots (100 μL) of the 10^−2^, 10^−4^, and 10^−6^ dilutions were spread onto LA plates (yeast extract 5.0 g, tryptone 10.0 g, NaCl 5.0 g, agar 15 g, distilled water 1000 mL, pH 7.0 ± 0.2), with sterile water as a negative control. Three replicate plates were used for each dilution, and the plates were incubated at 28 °C for 2 days. Single colonies with distinct morphological characteristics were selected, numbered, and purified.

For soil samples, 1 g of freshly collected soil was placed in a sterile centrifuge tube, mixed with 10 mL of sterile water, and incubated at 28 °C in a shaking incubator at 200 rpm for 4 h. After allowing the suspension to stand for 15 min, 100 μL of the supernatant was transferred to a sterile EP tube containing 900 μL of sterile water and serially diluted to 10^−6^. Aliquots (100 μL) of the 10^−2^, 10^−4^, and 10^−6^ dilutions were spread onto LA plates, using sterile water as a control. The plates were incubated at 28 °C for 2 days, and single colonies with distinct morphological characteristics were selected for purification. All purified strains were then transferred to LB liquid medium (same composition as LA but without agar) containing 40% glycerol and stored at −80 °C. Notes on strain naming: Strains prefixed with DR were isolated from the roots of wild *P. bulbocodioides*; strains prefixed with DS were isolated from rhizosphere soil samples of wild *P. bulbocodioides*; strains prefixed with DL were isolated from the leaves of wild *P. bulbocodioides*.

A 5 mm-diameter cork borer was used to prepare mycelial plugs of indicator pathogenic fungi 2-PT5 and 2-PT6, which were inoculated at the center of potato dextrose agar (PDA) plates. Each plate was divided evenly into four quadrants, and 1 μL of antagonistic bacterial suspension (OD_600_ = 1.0) was spot-inoculated onto three quadrants, with each inoculation spot 2 cm away from the central fungal plug. Plates spotted with an equivalent volume of LB liquid medium served as the blank control, and three biological replicates were set for each treatment. All plates were incubated upside down in a constant-temperature incubator at 28 °C under dark conditions. When the colony diameter of pathogenic fungi in the control group reached approximately three-quarters of the Petri dish diameter, the radial expansion diameter of pathogenic fungal colonies was measured along the transverse directions on both sides of the antagonistic bacterial spots with the fungal plug as the center. The antifungal inhibition rate of the bacterial strain was calculated using Equation 1.


Inhibitionrate(%)= (1)

$$\frac{\mathrm{Control}\,\mathrm{colony}\,\mathrm{diameter}-\mathrm{Treatment}\,\mathrm{colony}\,\mathrm{diameter}}{\mathrm{Control}\,\mathrm{colony}\,\mathrm{diameter}}\times 100$$

#### 2.3.2 Determination of siderophore and protease production capabilities of strain DR1

The abilities of strain DR1 to produce protease and siderophores were evaluated following the procedures described by Zhou et al. (2022), using specific media and experimental conditions as detailed in Supplementary Table 2. For each assay, five replicate plates were prepared per independent experiment, and the entire experiment was repeated three times (n = 3) for each activity index. A positive result was indicated by the formation of a clear zone around the colony. The diameters of the clear zone and the colony were measured, and the activity index was calculated using the following Equation 2 (Sharon et al., 2016; Florencio et al., 2012):


ActivityIndex=(Halozonediameter+ (2)

Colonydiameter)/Colonydiameter.

#### 2.3.3 Inhibition of pathogenic fungi by volatile organic compounds (VOCs) and cell-free filtrate of strain DR1

##### 2.3.3.1 VOC-mediated inhibition assay

Strain DR1 was inoculated into LB liquid medium and incubated overnight with shaking. Then, 10 μL of the culture was spotted onto the center of an LA solid agar plate. Separately, a 5-mm mycelial plug of the indicator fungi (strains 2-PT5 and 2-PT6) was placed in the center of a PDA plate. The LA plate and the PDA plate were placed face-to-face and sealed with Parafilm, allowing gas exchange only and preventing direct physical contact between the colonies (Ding et al., 2026). A control was prepared by spotting an equal volume of sterile LB medium onto an LA plate. All plates were incubated at 25 °C. When the mycelium of the control pathogen had grown to approximately three-quarters of the plate diameter, the colony diameter of the treated pathogen was measured, and the inhibition rate of mycelial growth by VOCs was calculated.

##### 2.3.3.2 Extracellular cell-free filtrate inhibition assay (Oxford cup method)

The overnight DR1 fermentation broth, obtained from the same batch used in the VOC experiments, was filtered through a 0.22-μm microporous membrane to obtain the sterile extracellular filtrate. The antifungal activity of the filtrate was evaluated using the Oxford cup agar diffusion method (Yang et al., 2025). Sterile Oxford cups were placed on solidified PDA plates at approximately one-third of the plate diameter, followed by the pouring of molten PDA (ca. 50 °C). After the agar had solidified, the cups were removed to create agar wells, into which 200 μL of the sterile DR1 filtrate was added, with an equal volume of sterile LB medium serving as the negative control. Mycelial plugs of the indicator fungi (strains 2-PT5 and 2-PT6) were inoculated on the opposite side of the plates at the corresponding one-third position. The plates were sealed and incubated at 28 °C. When the mycelial growth of the control pathogens reached three-quarters of the plate diameter, the colony radius of the treatment groups was measured, and the inhibition rate was calculated according to Equation 1. Furthermore, to assess the direct contact inhibitory effect of the filtrate, 1 mL of the aforementioned sterile filtrate was evenly spread onto the surface of solidified PDA plates. After the surface had slightly dried, mycelial plugs of the indicator fungi (strains 2-PT5 and 2-PT6) were placed at the center of the plates, while control plates were spread with sterile LB filtrate. The plates were sealed and incubated at 28 °C. Upon the mycelial growth of the control pathogens reaching three-quarters of the plate diameter, the colony diameters of the treatment groups were measured, and the inhibition rates were calculated using Equation 1. Each treatment consisted of three replicate plates, and the entire experiment was performed in triplicate. The results are expressed as the mean ± standard deviation (SD).

#### 2.3.4 Hyphal morphological observation under different antagonistic treatments

To observe the growth morphology of inhibited hyphae, the dual-culture confrontation assay plates, VOC inhibition assay plates, and blank control plates prepared from the same batch as described in Sections 2.3.1 and 2.3.3 were observed and photographed under a microscope. Morphological changes were recorded and compared with the control. For the recovery assay, the VOC treatment system was terminated after 5 days, the VOC source was removed, and the pathogen plates were further incubated for 5 days to observe hyphal regrowth.

#### 2.3.5 Identification of biocontrol bacteria

After selection of strain DR1, which exhibited the strongest antagonistic activity for further characterization, genomic DNA was extracted using the FastPure^®^ Bacterial Genomic DNA Extraction Kit (Vazyme, Nanjing, China). Universal primers were used to amplify the 16S ribosomal RNA (16S rRNA) gene (27F/1492R; Weisburg et al., 1991), and specific primers were used to amplify the DNA gyrase subunit B (*gyrB*) (UP1/UP2r; Yamamoto and Harayama, 1995), RNA polymerase subunit beta (*rpoB*) (LAPS/LAPS27; Ait Tayeb et al., 2005), and RNA polymerase sigma factor RpoD (*rpoD*) (PsEG30F/PsEG790R; Mulet et al., 2009) genes. The PCR reaction mixture composition was as described in Section 2.2.4, and the amplification programs were as described in the corresponding references. After sequencing, the obtained sequences were assembled using SeqMan software and subjected to BLAST homology searches in the NCBI database. All phylogenetic analyses were performed in PhyloSuite v1.2.3 (Zhang et al., 2020). Phylogenetic reconstruction was performed as described in Section 2.2.5.

### 2.4 *In vivo* control efficacy of DR1 against *P. bulbocodioides* anthracnose

#### 2.4.1 Indoor pot experiment for leaf control

Seedlings of the same batch and size were obtained from Guangnan Yaowanggu Biotechnology Co., Ltd. One hundred seedlings were transplanted into peat soil sterilized at 121 °C for 30 min and watered with sterile water. They were maintained at 25 ± 2 °C, 70–80% relative humidity, and a 12 h light/dark cycle. For the leaf control experiment against *P. bulbocodioides* anthracnose, 36 healthy plants were selected. Leaves were wiped with sterile water, air-dried, disinfected with 75% ethanol, and then wounded twice per leaf using a sterile needle. Each treatment consisted of 3 replicates (n = 3), with each replicate comprising 3 pots (9 pots per treatment in total). Four treatments were applied: (i) prevention (DR1 suspension at OD_600_ = 1.0 sprayed 24 h before inoculation with strain 2-PT6); (ii) treatment (the same DR1 spray applied immediately after inoculation); (iii) positive control (inoculation with 2-PT6 alone); and (iv) negative control (spraying with DR1 alone without pathogen). Pathogen inoculation was performed by placing 4-mm fungal plugs taken from 7-day-old cultures onto the wounds. Leaves were moistened with sterile water at 4 h post-inoculation. Disease investigation was conducted 12 days after pathogen inoculation. Each biological replicate (3 potted plants) served as an individual statistical unit, yielding three independent datasets of disease incidence and lesion length per treatment. Disease incidence was calculated as the total number of lesions on all leaves divided by the total number of inoculated wounds (3 leaves per plant × 2 wounds per leaf, i.e., 6 wounds per replicate). Lesion length was measured with a straight ruler, and the average length of all leaves within a replicate was recorded as its lesion length value.

#### 2.4.2 Greenhouse experiment on natural disease

The greenhouse experiment was conducted at the *P. bulbocodioides* cultivation base of Guangnan Yaowanggu Biotechnology Co., Ltd. in Wenshan Prefecture, Yunnan. Anthracnose had naturally occurred on nursery beds after leaf emergence from tissue-cultured pseudobulbs. Six beds (1.5 m × 2.0 m each) with similar disease severity were arranged in a randomized block design, with three beds assigned to the treatment group (DR1 spray) and three to the control group (water only). Based on the application methods reported in previous studies (e.g., 100-fold dilution of *P. fluorescens* in Shen et al., 2014; 300-fold dilution of *B. velezensis* AP6 fermentation broth in Li et al., 2026) and the practical considerations of field operability, cost-effectiveness, and crop safety, the overnight culture of DR1 (OD_600_ = 1.8) was diluted 100-fold with sterile water and applied at 0.33 L/m^2^ once per month in the greenhouse experiment. Control beds were given an equal volume of sterile water. All beds were watered every 3 days, and other agronomic practices (e.g., weeding) were applied uniformly to both groups.

Disease assessment was performed 4 months after the first spray. Five quadrats (0.5 m × 0.5 m) were randomly placed in each bed, resulting in 15 quadrats per treatment. Disease status and the number of newly emerged leaves were recorded for each quadrat. For leaf length measurement, 15 leaves were randomly selected from all leaves collected across the 15 quadrats of each treatment. Leaf disease severity was graded as follows: Grade 0, no lesions; Grade 1, lesion area covering < 30% of the total leaf area; Grade 2, lesion area covering 30-50%; Grade 3, lesion area covering > 50%. The disease index was calculated using the Equation 3 of Chiang et al. (2017):


Disease⁢index%= (3)

$$\frac{\sum \left(\mathrm{Number}\,\mathrm{of}\,\mathrm{diseased}\,\mathrm{leaves}\,\mathrm{at}\,\mathrm{each}\,\mathrm{grade}\times \mathrm{Grade}\,\mathrm{value}\right)}{\mathrm{Total}\,\mathrm{number}\,\mathrm{of}\,\mathrm{leaves}\,\mathrm{investigated}\times \mathrm{Highest}\,\mathrm{grade}\,\mathrm{value}}\times 100$$

Control efficacy was calculated by first analyzing the raw disease index values with a beta regression model (Mathew et al., 2026) in R software, and then substituting the estimated disease proportions from the model into Equation 4 (Piepho et al., 2024).


$$\epsilon =\left(1-\frac{\mu_{t}}{\mu_{c}}\right)\times 100$$ (4)

where: μ_*t*_ is the Beta-regression-fitted mean disease index of the treatment group; μ_*c*_ is the Beta-regression-fitted mean disease index of the control group.

### 2.5 Data statistics

For the morphological observations (colony diameter, sporulation, spore size, and length-to-width ratio in Section 2.2.3) and the VOC/cell-free filtrate inhibition assays (Section 2.3.3), data were first checked for normality and homogeneity of variance. Data satisfying these assumptions were compared using independent-sample *t*-tests, whereas non-normally distributed data were compared using Wilcoxon rank-sum tests (*P* < 0.05). Colony diameters were compared between the two strains at each time point using Welch’s independent-sample *t*-tests (n = 3); because four time points were compared, Bonferroni correction was applied and the significance threshold was set at α = 0.05/4 = 0.0125. No sporulation was detected on day 3, so day 3 was excluded from statistical testing. On day 5, all replicates of strain 2-PT5 were zero, whereas all replicates of strain 2-PT6 were positive; therefore, the spore count of 2-PT6 was compared with zero using a one-sample *t*-test. On days 8, 10, 13, and 15, independent-sample t-tests were used to compare spore production between the two strains (n = 5 per strain); because five time points were compared, Bonferroni correction was applied and the significance threshold was set at α = 0.05/5 = 0.01. Spore size and length-to-width ratio were analyzed using each spore as the observational unit (n = 150 per strain) with Wilcoxon rank-sum tests. For the VOC/cell-free filtrate inhibition assays, comparisons between the two groups were performed using independent-sample t-tests or Wilcoxon rank-sum tests, as appropriate. All analyses were performed using SPSS 26.0.

In the greenhouse experiment (Section 2.4.2), three beds were assigned to each treatment, and five quadrats were randomly surveyed within each bed, giving 15 quadrats per treatment. For the disease index (DI), number of newly emerged leaves, average leaf length, and number of new seedlings used for boxplot visualization and descriptive comparison, each quadrat was used as the observational unit (n = 15 per treatment). Comparisons between the treatment and control groups were performed using independent-sample *t*-tests (*P* < 0.05) in SPSS 26.0. The DI used to calculate control efficacy was calculated as the weighted sum of the numbers of leaves in disease grades 0, 1, 2, and 3, and is a continuous proportional variable on the (0,1) interval. The DI of each quadrat was calculated according to Equation 3 (Chiang et al., 2017), yielding 15 quadrat-level DI values per treatment. Because the data contained boundary values (0), an extended-support beta regression was used to model the DI (Mathew et al., 2026). All analyses were performed in R software (version 4.6.0). Models were fitted using the betareg package (version 3.1-4), and marginal means and pairwise comparisons among treatments were estimated using the emmeans package (version 1.8.0), with treatment comparisons performed using Wald tests. The estimated marginal means were then substituted into Equation 4 to calculate control efficacy (Piepho et al., 2024).

For the *in vitro* fungicide toxicity assays (Section 2.2.6), toxicity regression equations were established, and the EC_50_ and EC_95_ values with their 95% confidence intervals were calculated using DPS 9.5 software.

## 3 Results

### 3.1 Isolation and identification of the pathogen

#### 3.1.1 Pathogenicity tests and fulfillment of Koch’s postulates

Using the tissue isolation method, a total of 10 fungal isolates were obtained from diseased *P. bulbocodioides* samples (7 from pseudobulbs and 3 from leaves). Pathogenicity tests showed that inoculation of strain 2-PT6 (isolated from diseased pseudobulbs and leaves; Figures 1a–c,e) onto leaves resulted in lesions similar to those of diseased samples on all inoculated leaves at 5 days post-inoculation (Figure 1e); inoculation of strain 2-PT5 (isolated from diseased pseudobulbs; Figures 1b,d,g) and strain 2-PT6 onto pseudobulbs produced similar lesions on all inoculated pseudobulbs at 10 days post-inoculation (Figures 1f,g). The remaining two strains isolated from leaves were completely non-pathogenic on leaves, and the other five strains isolated from pseudobulbs showed no lesion expansion on pseudobulbs. From the diseased tissues inoculated with the strains, re-isolations were performed, and from each respective tissue one strain with colony morphology similar to the original inoculation strain was obtained. The original isolates and the re-isolated strains were subjected to *ITS* sequencing, and pairwise sequence alignments using NCBI BLAST (blastn) confirmed that the *ITS* sequences of the re-isolates from both tissues shared 100% identity with those of the respective original inoculation strains (see Supplementary Figures 1–3 for sequence alignments). The multilocus sequences of the original isolates were deposited in GenBank (accession numbers are listed in Section 3.1.3). Koch’s postulates were thus fulfilled, confirming that strain 2-PT5 (isolated from pseudobulbs) and strain 2-PT6 (isolated from both pseudobulbs and leaves) are the causal agents of the disease.

**FIGURE 1:**
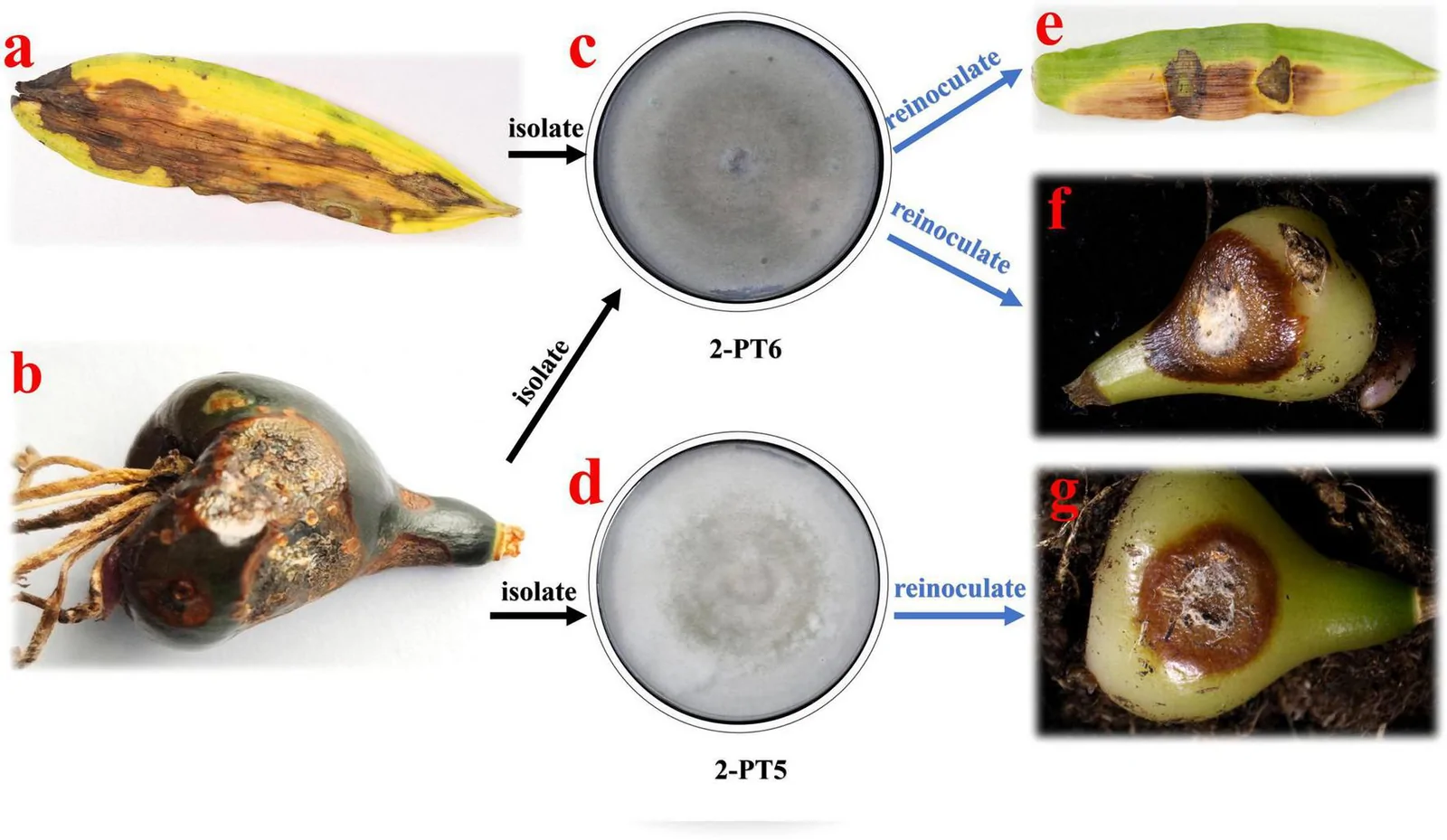
Isolation and re-inoculation verification of the pathogen causing *P. bulbocodioides* anthracnose. **(a)** Diseased leaf from a plantation; **(b)** Diseased pseudobulb from a plantation; **(c)** Strain 2-PT6 isolated from leaf and pseudobulb; **(d)** Strain 2-PT5 isolated from pseudobulb; **(e)** Leaf re-inoculated with 2-PT6; **(f)** Pseudobulb re-inoculated with strain 2-PT6; **(g)** Pseudobulb re-inoculated with strain 2-PT5.

#### 3.1.2 Morphological characterization and comparison of strains 2-PT5 and 2-PT6

Both strains formed grayish-white colonies with pale grayish-green centers on PDA medium (Figures 1c,d), but visually, strain 2-PT6 grew faster and developed the central grayish-green color earlier. Microscopic observation revealed that both strains possessed septate, branched, colorless to pale gray hyphae forming a network (Figures 2a,c). Conidia were single-celled, oblong to ellipsoid, pale brown, and borne on phialidic conidiogenous cells (Figures 2b,d). As shown in Figure 3a, Strain 2-PT6 grew faster than strain 2-PT5 on PDA plates during the early stage. At day 3, the colony diameter of strain 2-PT6 was significantly larger than that of strain 2-PT5 (mean difference = 4.34 mm, *P* = 0.00016). At day 5, 2-PT6 remained significantly larger than 2-PT5 (mean difference = 5.23 mm, *P* = 0.00696). At day 8, no significant difference was detected between the two strains (mean difference = 2.89 mm, *P* = 0.293). At day 10, 2-PT6 was again significantly larger than 2-PT5 (mean difference = 3.39 mm, *P* = 0.00385). After Bonferroni correction, the differences at days 3, 5, and 10 remained significant (*P* < 0.0125), whereas the difference at day 8 did not.

**FIGURE 2:**
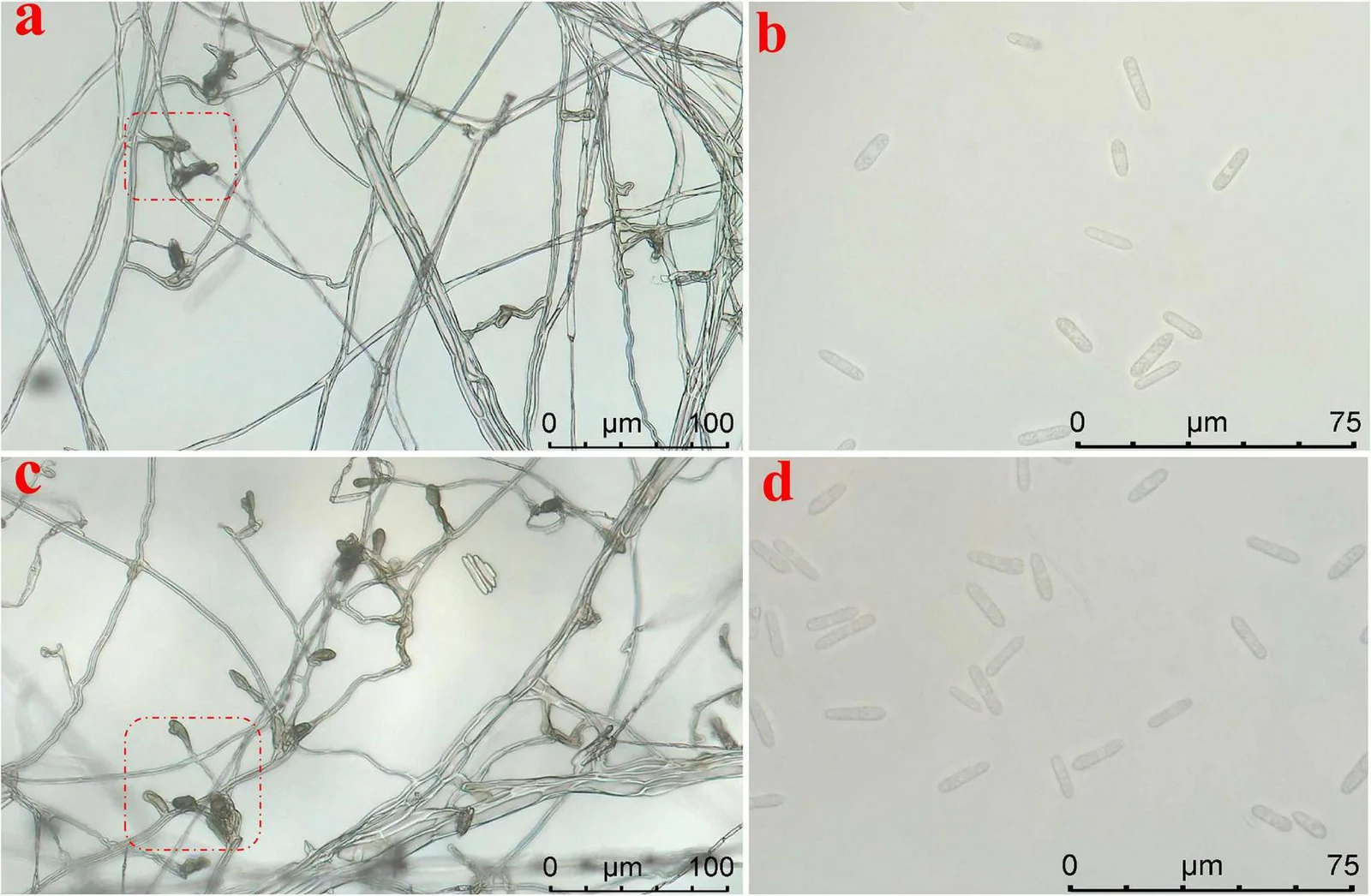
Comparison of microscopic morphology between the two pathogenic strains. **(a)** Hyphae and conidiophores (as shown in the red dashed box) of strain 2-PT5; **(b)** Conidia of strain 2-PT5; **(c)** Hyphae and conidiophores (as shown in the red dashed box) of strain 2-PT6; **(d)** Conidia of strain 2-PT6.

**FIGURE 3:**
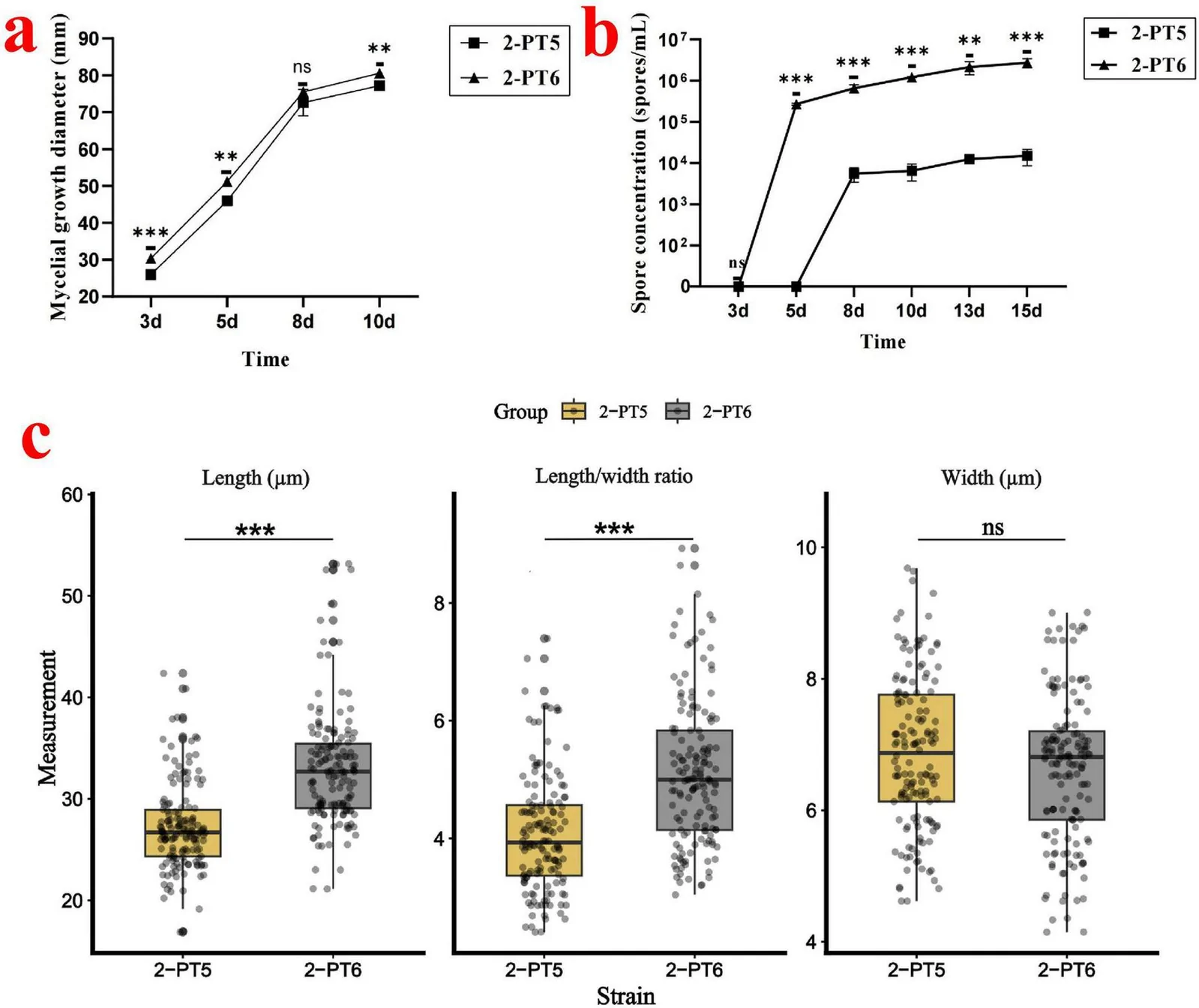
Comparison of morphological characteristics between strains 2-PT5 and 2-PT6. **(a)** Colony growth dynamics of the two strains on PDA medium over 10 days. Significance was determined by Welch’s independent-sample *t*-tests at each time point with Bonferroni correction (α = 0.0125). **(b)** Sporulation dynamics of the two strains on PDA medium over 15 days. Spore counts are presented as log_10_ (spores/mL + 1) to accommodate zero values; zero values indicate no sporulation detected. Significance was determined by one-sample *t*-test (day 5) or independent-sample *t*-tests (days 8–15) with Bonferroni correction (α = 0.01). **(c)** Comparison of conidial morphology and dimensions between the two strains. Data are presented as mean ± standard deviation (SD). Statistical significance was determined by Wilcoxon rank-sum tests. Significance levels: **P < 0.01, ***P < 0.0001; ns, not significant.

As shown in Figure 3b, The sporulation capacity of strain 2-PT6 was significantly higher than that of strain 2-PT5. No sporulation was detected for either strain on day 3. On day 5, all replicates of strain 2-PT5 remained at zero, whereas strain 2-PT6 produced 2.70 × 10^5^ spores/mL (one-sample *t*-test against zero, *P* = 1.33 × 10^−6^). From day 8 onward, strain 2-PT6 produced significantly more spores than strain 2-PT5 at all time points (day 8: *P* = 5.15 × 10^−4^; day 10: *P* = 4.17 × 10^−8^; day 13: *P* = 2.98 × 10^−3^; day 15: *P* = 8.68 × 10^−4^; independent-sample t-tests). After Bonferroni correction (α = 0.05/5 = 0.01), all these differences remained significant. The most pronounced difference was observed on day 10 (*P* < 0.0001). The spore yield of 2-PT5 increased slowly, reaching a maximum of only approximately 1.5 × 10^4^ spores/mL. In contrast, 2-PT6 exhibited exponential growth, attaining 2.75 × 10^6^ spores/mL by day 15, which was roughly 183-fold higher than that of 2-PT5. As shown in Figure 3c, Strain 2-PT5 produced conidia measuring (16.9–42.4) × (4.62–9.69) μm (mean ± SD: 27.20 ± 4.25 × 6.88 ± 1.16 μm, n = 150), with a length-to-width ratio of 4.08 ± 1.00. In contrast, strain 2-PT6 produced conidia measuring (21.1–53.2) × (4.14–9.01) μm (mean ± SD: 33.04 ± 5.80 × 6.64 ± 1.13 μm, n = 150), with a length-to-width ratio of 5.13 ± 1.24. Both strains produced cylindrical to ellipsoid conidia with obtuse ends. Wilcoxon rank-sum tests revealed that strain 2-PT6 had significantly longer conidia (*P* < 0.001) and a significantly higher length-to-width ratio (*P* < 0.001), whereas conidial width did not differ significantly between the two strains (*P* = 0.135).

#### 3.1.3 Molecular identification of strain 2-PT5 and 2-PT6

Multilocus sequence analysis using *ITS*, *CHS, TUB2, GAPDH*, and *ACT* genes placed both 2-PT5 and 2-PT6 within the clade of *C. orchidophilum* reference strain CBS 632.80 with 100% bootstrap support (Figure 4). The NCBI GenBank accession numbers for the sequences of strain 2-PT5 are as follows: *ITS* (PZ463163), *CHS* (PZ464322), *ACT* (PZ464323), *TUB2* (PZ464324), and *GAPDH* (PZ467636). For strain 2-PT6, the accession numbers are *ITS* (PZ463164), *CHS* (PZ464559), *ACT* (PZ464561), *TUB2* (PZ464562), and *GAPDH* (PZ464560). Based on morphological and molecular evidence, both strains were identified as *C. orchidophilum*. This study reports the occurrence of anthracnose caused by *C. orchidophilum* on *P. bulbocodioides*, and reveals that morphologically differentiated strains of this pathogen exist on the same host.

**FIGURE 4:**
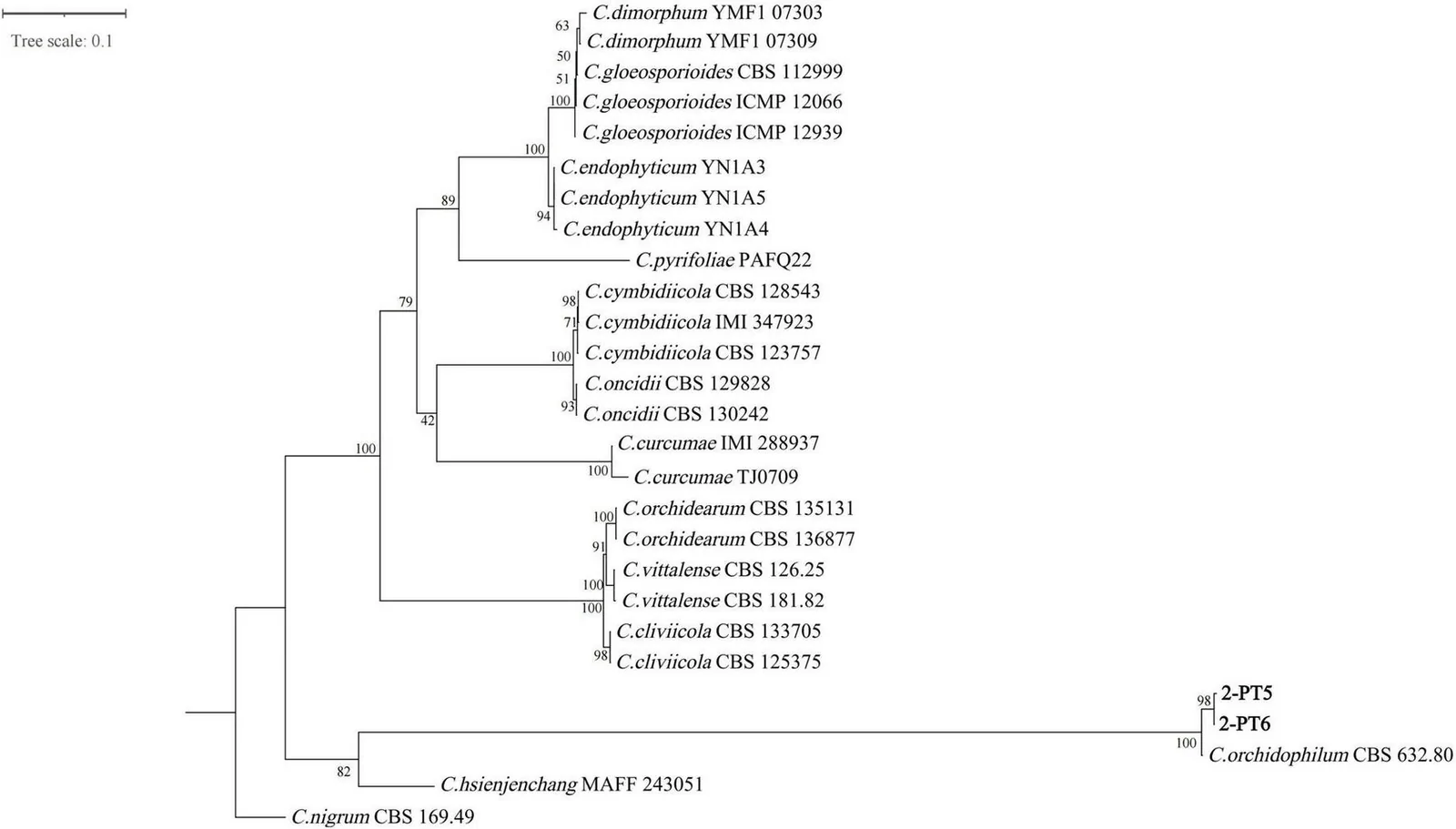
Phylogenetic tree of 2-PT5 and 2-PT6 based on multilocus sequences (*ITS*, *CHS*, *TUB2*, *GAPDH*, *ACT*) using Maximum Likelihood. Bootstrap values (1000 replicates) shown.

#### 3.1.4 *In vitro* toxicity of fungicides against *C. orchidophilum*

The toxicity of the six tested fungicides against the two *C. orchidophilum* strains is summarized in Table 1. Among them, 25% pyraclostrobin exhibited the strongest activity, with EC_50_ values of 0.0060 mg/L against 2-PT6 and 0.0031 mg/L against 2-PT5, which were 1–5 orders of magnitude lower than those of the other fungicides. Difenoconazole (250 g/L) and prochloraz (450 g/L) also displayed strong inhibitory toxicity toward the pathogen. In contrast, copper sulfate and fludioxonil showed poor inhibitory performance, with EC_50_ values exceeding 180 mg/L.

**TABLE 1:** Toxicity of six fungicides against *C. orchidophilum* strains 2-PT5 and 2-PT6.

Fungicide	Strain	Toxicity regression equation	Intercept (A)	Slope (B)	Correlation coefficient (R^2^)	EC_50_ (mg/L) (95% CI)	EC_95_ (mg/L)
25 g/L fludioxonil SC	2-PT6	y = 3.9296 + 0.4094x	3.9296	0.4094	0.9649	411.5 (89.2 1898.1)	4.29 × 106
	2-PT5	y = 3.8949 + 0.4658x	3.8949	0.4658	0.9283	235.6 (31.0 1792.2)	8.02 × 105
90% carbendazim WP	2-PT6	y = 4.7542 + 0.7234x	4.7542	0.7234	0.818	2.19 (0.52 9.27)	411
	2-PT5	y = 4.4133 + 0.7489x	4.4133	0.7489	0.9481	6.07 (2.40 15.37)	954.3
100 g/L copper sulfate AS	2-PT6	y = −5.8669 + 4.8080x	−5.8669	4.808	0.8024	182.0 (31.9 1038.6)	399.3
	2-PT5	y = −7.4471 + 5.3004x	−7.4471	5.3004	0.8105	223.0 (47.8 1040.5)	456
25% pyraclostrobin SC	2-PT6	y = 5.8471 + 0.3812x	5.8471	0.3812	0.99	0.0060 (0.0027 0.0134)	123.8
	2-PT5	y = 5.9525 + 0.3798x	5.9525	0.3798	0.9818	0.0031 (0.0009 0.0109)	66.48
450 g/L prochloraz EW	2-PT6	y = 6.4936 + 2.1196x	6.4936	2.1196	0.9587	0.1974 (0.1013 0.3845)	1.18
	2-PT5	y = 5.7484 + 0.9249x	5.7484	0.9249	0.8597	0.16 (0.04 0.63)	9.24
250 g/L difenoconazole EC	2-PT6	y = 7.0617 + 1.9013x	7.0617	1.9013	0.9407	0.0823 (0.0340 0.1993)	0.6
	2-PT5	y = 5.9177 + 1.1305x	5.9177	1.1305	0.9435	0.15 (0.07 0.35)	4.38

### 3.2 Isolation, screening, and identification of biocontrol bacteria

#### 3.2.1 Isolation and preliminary screening of antagonistic strains

Isolation and antagonistic screening. A total of 6, 6, and 3 culturable bacterial strains were isolated from the roots (endophytic), rhizosphere soil, and leaves of *P. bulbocodioides*, respectively. The antagonistic activity of these strains was evaluated using the dual culture method (Figure 5). Among all isolates tested, the 4 strains with the highest inhibition rates (DR1, DS1, DR4, DS3) were all derived from roots or rhizosphere soil, whereas strains isolated from leaves showed weak or no antagonistic activity. Notably, strong antagonistic activity was exhibited by strain DR1, isolated from the root endosphere of *P. bulbocodioides* (Figure 6a), against the target strains (2-PT5 and 2-PT6), with inhibition rates of 55.82 ± 1.76% against 2-PT5 and 71.28 ± 0.84 % against 2-PT6 (Figures 5a,b). During dual-culture incubation, orange-red conidial masses appeared at the margins of pathogen hyphae inhibited by DR1 (Figures 6c,d) (Li et al., 2023).

**FIGURE 5:**
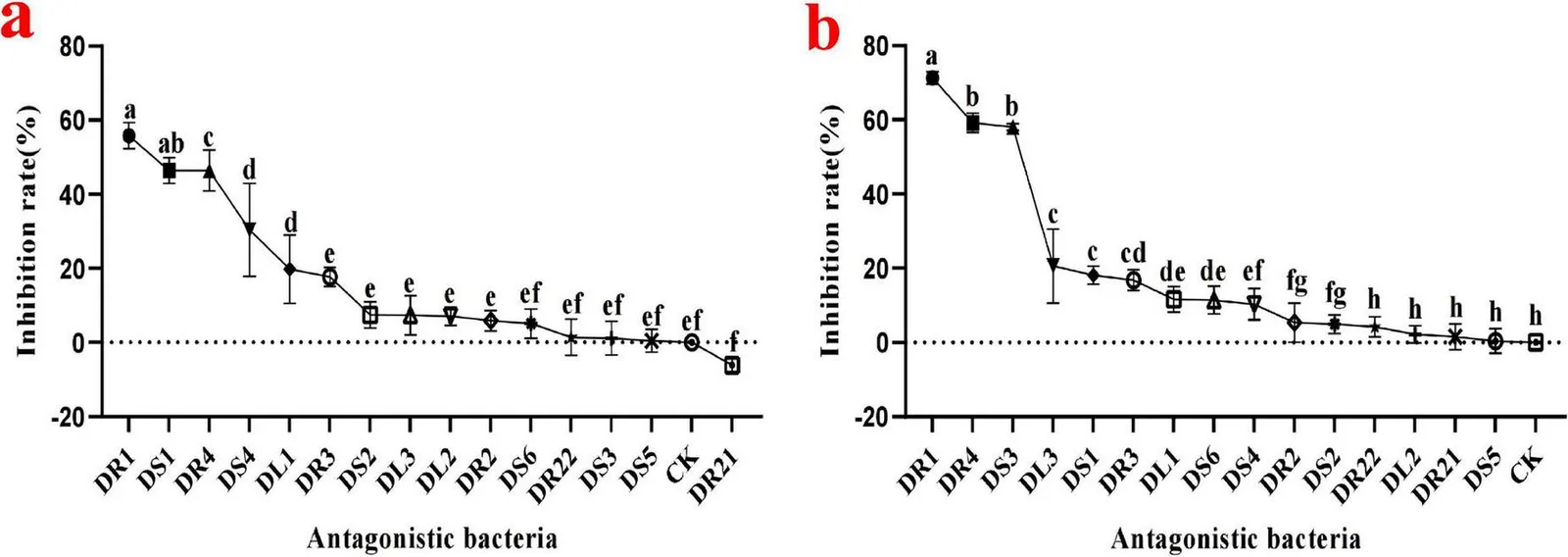
Inhibition rates of strains isolated from the rhizosphere and root interior of *P. bulbocodioides* against pathogens. **(a)** Against *C. orchidophilum* strain 2-PT5; **(b)** Against *C. orchidophilum* strain 2-PT6. Notes on strain naming: Strains prefixed with DR were isolated from the roots of wild *P. bulbocodioides*; strains prefixed with DS were isolated from rhizosphere soil samples of wild *P. bulbocodioides*; strains prefixed with DL were isolated from the leaves of wild *P. bulbocodioides*.

**FIGURE 6:**
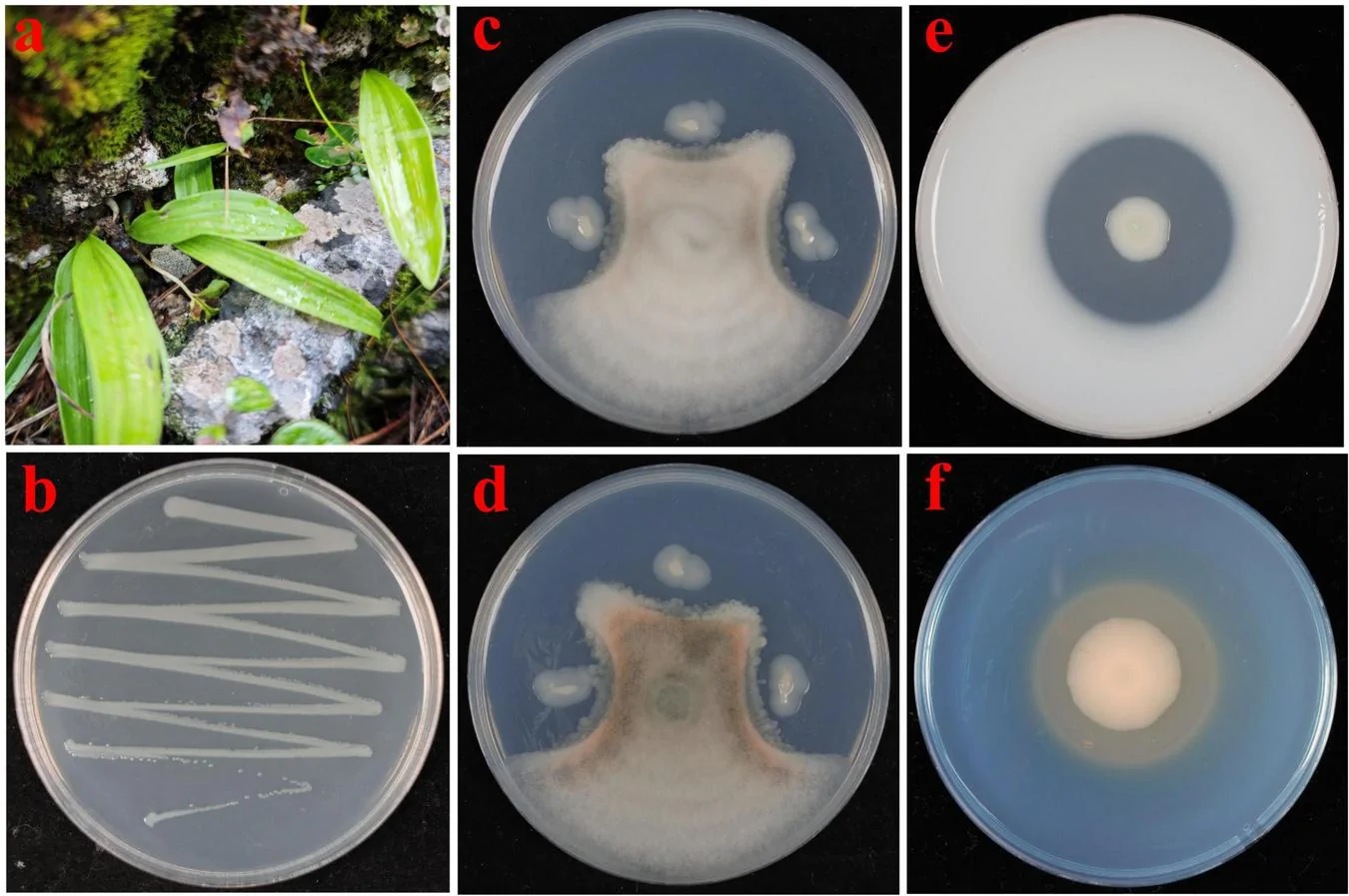
Isolation and antagonistic activity of strain DR1. **(a)** DR1 isolated from wild *P. bulbocodioides* root; **(b)** Colony morphology on LA plate; **(c)** Antagonistic effect against *C. orchidophilum* 2-PT5; **(d)** Against *C. orchidophilum* 2-PT6; **(e)** Protease activity; **(f)** Siderophore production.

#### 3.2.2 Quantitative determination of protease and siderophore activities of strain DR1

Strain DR1 formed obvious clear zones on the indicator plates for protease and siderophore production. The results showed that the protease activity index averaged 3.044 with a standard deviation of 0.366, and the values ranged from 2.263 to 3.719 (Figure 6e); the siderophore activity index averaged 1.792 with a standard deviation of 0.120, and the values ranged from 1.513 to 1.983 (Figure 6f).

#### 3.2.3 Inhibition of pathogenic fungi by volatile organic compounds (VOCs) and cell-free filtrate of strain DR1

As shown in Figure 7a, the VOC inhibition assay demonstrated that the volatile organic compounds (VOCs) produced by strain DR1 significantly inhibited the mycelial growth of pathogenic fungi 2-PT5 and 2-PT6 (independent-sample *t*-test, *P* < 0.01). Compared with the control, the colony diameter of 2-PT5 co-cultured with DR1 decreased from 72.71 ± 3.70 mm to 34.14 ± 2.19 mm, with an inhibition rate of 53.04 ± 3.02%; for 2-PT6, the colony diameter decreased from 75.51 ± 0.87 mm to 39.20 ± 2.75 mm, corresponding to an inhibition rate of 48.08 ± 3.65%. In the cell-free extracellular filtrate treatment group, no significant difference in mycelial growth was observed for either pathogen compared with the control, indicating that the extracellular products of DR1 exerted no direct inhibitory effect on 2-PT5 and 2-PT6 under cell-free conditions (Figure 7b).

**FIGURE 7:**
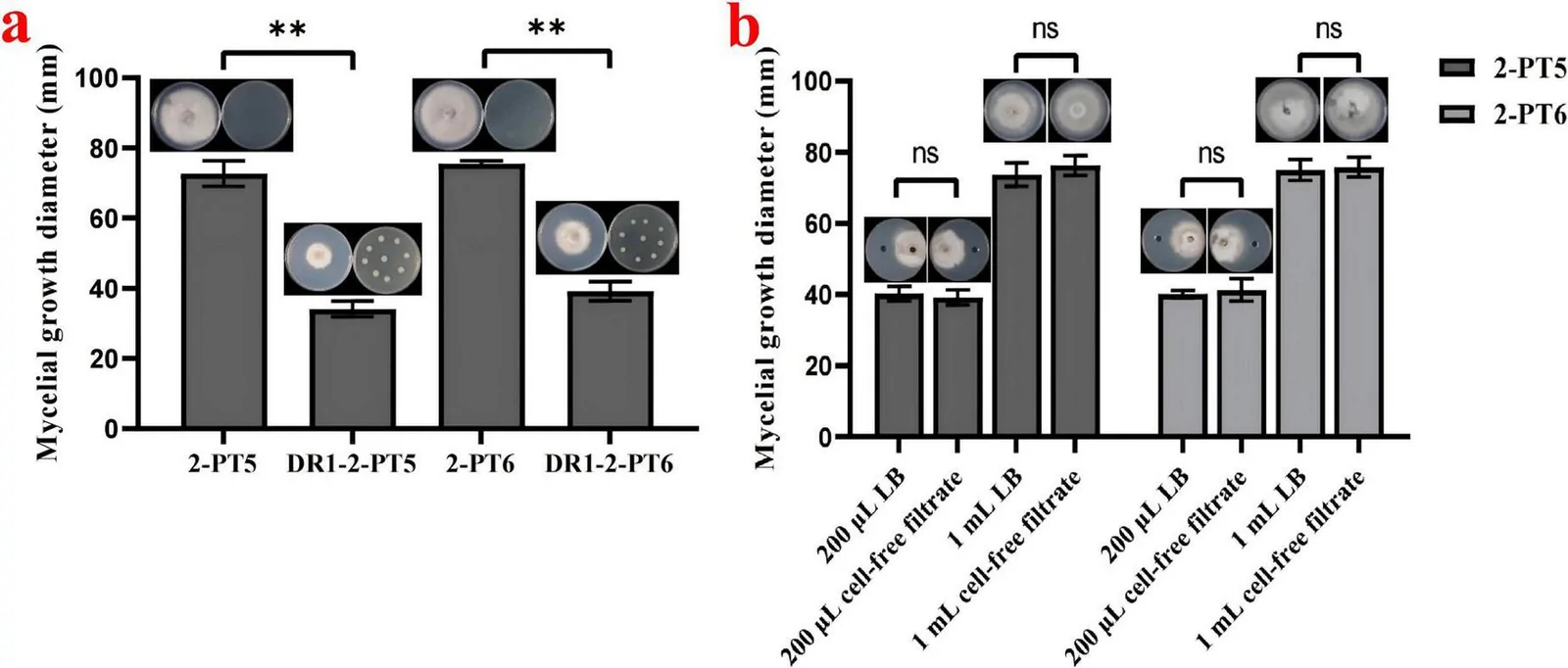
Effects of volatile organic compounds (VOCs) and cell-free filtrate from strain DR1 on mycelial growth of pathogenic fungi 2-PT5 and 2-PT6. **(a)** VOC inhibition assay. **(b)** Cell-free extracellular filtrate inhibition assay. Data are presented as mean ± SD; statistical significance: **P < 0.01, ns: not significant.

#### 3.2.4 Hyphal morphological changes of pathogenic isolates under different antagonistic treatments

As shown in Figure 8, under external stress conditions, the two pathogenic fungal isolates (2-PT5 and 2-PT6) exhibited consistent responses. Compared with the control (Figures 8a,b), in the plate confrontation assay where the fungal colonies and the biocontrol strain DR1 were physically separated, the hyphal tips facing DR1 showed pronounced growth arrest, and radial extension from the colony margin was significantly inhibited. Concomitantly, excessive and abnormal lateral branching occurred, resulting in the formation of highly branched coral-like or tree-like clustered structures (Figures 8c,d). In contrast, when only volatile organic compounds (VOCs) were allowed to interact in a dual-culture system, VOC exposure markedly suppressed the growth and biomass accumulation of marginal aerial hyphae, which became curled and knotted (Figures 8e,f). Additionally, hyphal lysis and leakage of intracellular contents were observed (Figures 8g,h). Notably, after terminating the dual culture and removing VOCs for five days, some aerial hyphae resumed growth (Figures 8i–l). The cell-free fermentation filtrate of strain DR1 showed no obvious inhibitory effect on hyphal growth, nor did it induce any significant morphological abnormalities.

**FIGURE 8:**
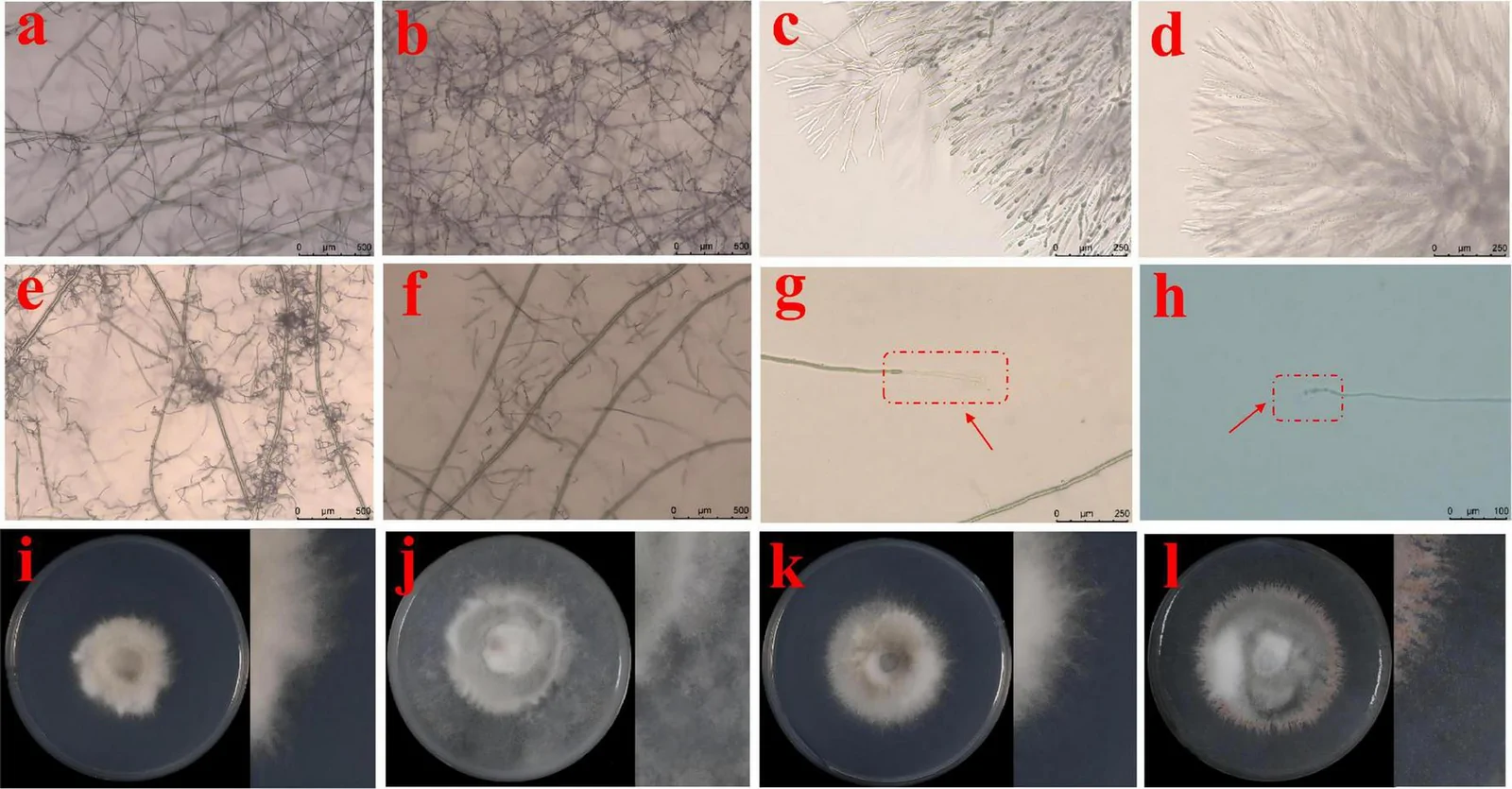
Morphological responses of pathogenic fungal isolates (2-PT5 and 2-PT6) to biocontrol strain DR1 under different assay conditions. **(a, b)** Controls: normal hyphal growth of isolates 2-PT5 and 2-PT6, respectively, without any treatment; **(c, d)** Plate confrontation assay: coral-like or tree-like clustered structures formed at the hyphal tips of 2-PT5 **(c)** and 2-PT6 **(d)** facing DR1; **(e, f)** VOC exposure: aerial hyphae of 2-PT5 **(e)** and 2-PT6 **(f)** became reduced, curled, and knotted; **(g, h)** VOC exposure showing hyphal lysis and leakage of intracellular contents in 2-PT5 **(g)** and 2-PT6 **(h)** (indicated by arrowheads); **(i–l)** Recovery after VOC removal: some aerial hyphae resumed growth in both 2-PT5 **(i, j)** and 2-PT6 **(k, l)** 5 days after terminating the dual culture.

#### 3.2.5 Identification and phylogenetic analysis of strain DR1

Identification of strain DR1. On LA medium, circular, smooth-edged, opaque, cream-colored colonies with a buttery texture were formed by strain DR1 (Figure 6b). Phylogenetic analysis based on 16S rRNA gene and three housekeeping genes *(gyrB*, *rpoB*, *rpoD*) placed DR1 within a highly supported clade (100% bootstrap) together with the reference strains F77 and 1008 of *P. pergaminensis* (Figure 9). The NCBI GenBank accession numbers for the sequences of strain DR1 are as follows: 16S rRNA (PZ460984), *gyrB* (PZ464321), *rpoB* (PZ464557), and *rpoD* (PZ464558). Therefore, strain DR1 was identified as *P. pergaminensis*.

**FIGURE 9:**
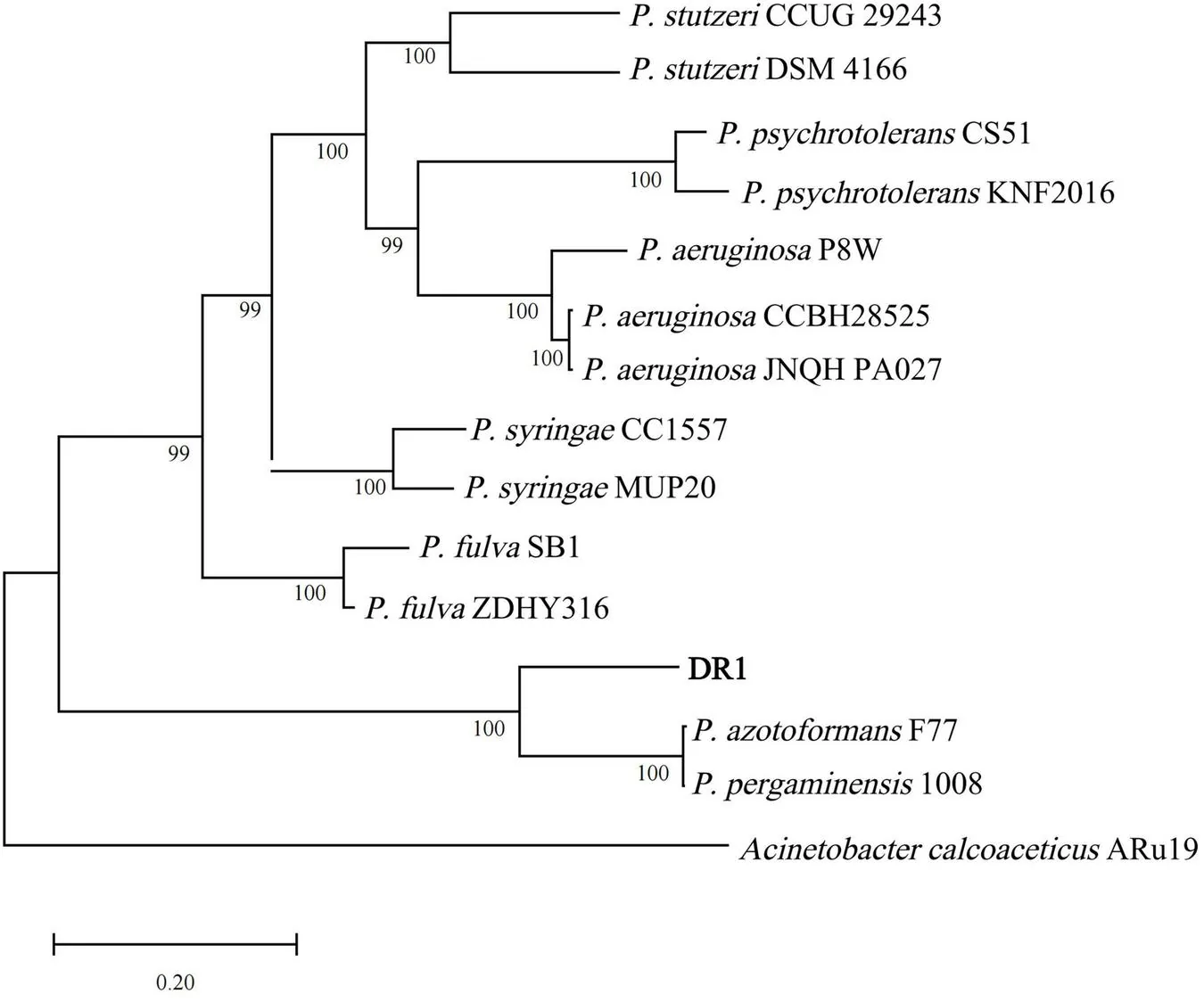
Phylogenetic tree of DR1 based on 16S rRNA, *gyrB*, *rpoB*, and *rpoD* sequences using Maximum Likelihood. Bootstrap values (1000 replicates) are shown at nodes.

### 3.3 Control efficacy of *P. pergaminensis* DR1 against *P. bulbocodioides* anthracnose

#### 3.3.1 Indoor pot experiment for leaf control

As shown in Figure 10a, the positive control (inoculation with 2-PT6 alone) showed obvious anthracnose symptoms on *P. bulbocodioides* leaves. In comparison, as shown in Figure 10b, the negative control (spraying with DR1 alone without pathogen) showed that *P. pergaminensis* DR1 treatment caused no adverse effects on plant health, while the other groups developed disease symptoms to varying degrees. As shown in Figures 10e,f, 12 days after pathogen inoculation, compared with the positive control group (Figure 10a; lesion length 3.89 ± 0.08 cm, disease incidence 100%), the treatment group (Figure 10c; the same DR1 spray applied immediately after inoculation) also exhibited a lesion length significantly lower than that of the control (2.68 ± 0.34 cm, *P* < 0.05), but its disease incidence (94.44 ± 5.56%) was not significantly different from the control (100%) (*P* > 0.05). The prevention group (Figure 10d; DR1 suspension at OD_600_ = 1.0 sprayed 24 h before inoculation with strain 2-PT6) showed significantly reduced lesion length (1.49 ± 0.38 cm) and disease incidence (66.67 ± 9.62%) (*P* < 0.05), indicating that the preventive treatment effectively suppressed both lesion expansion and disease incidence. These results demonstrate that the preventive treatment effectively reduces both disease occurrence and lesion expansion, whereas the curative treatment only partially inhibits lesion expansion without significantly improving disease incidence. It should be noted that at 15 days post-inoculation, all diseased leaves in the curative treatment group exhibited complete rot, while the diseased leaves of the prevention group developed total necrosis and rot at 18 days post-inoculation.

**FIGURE 10:**
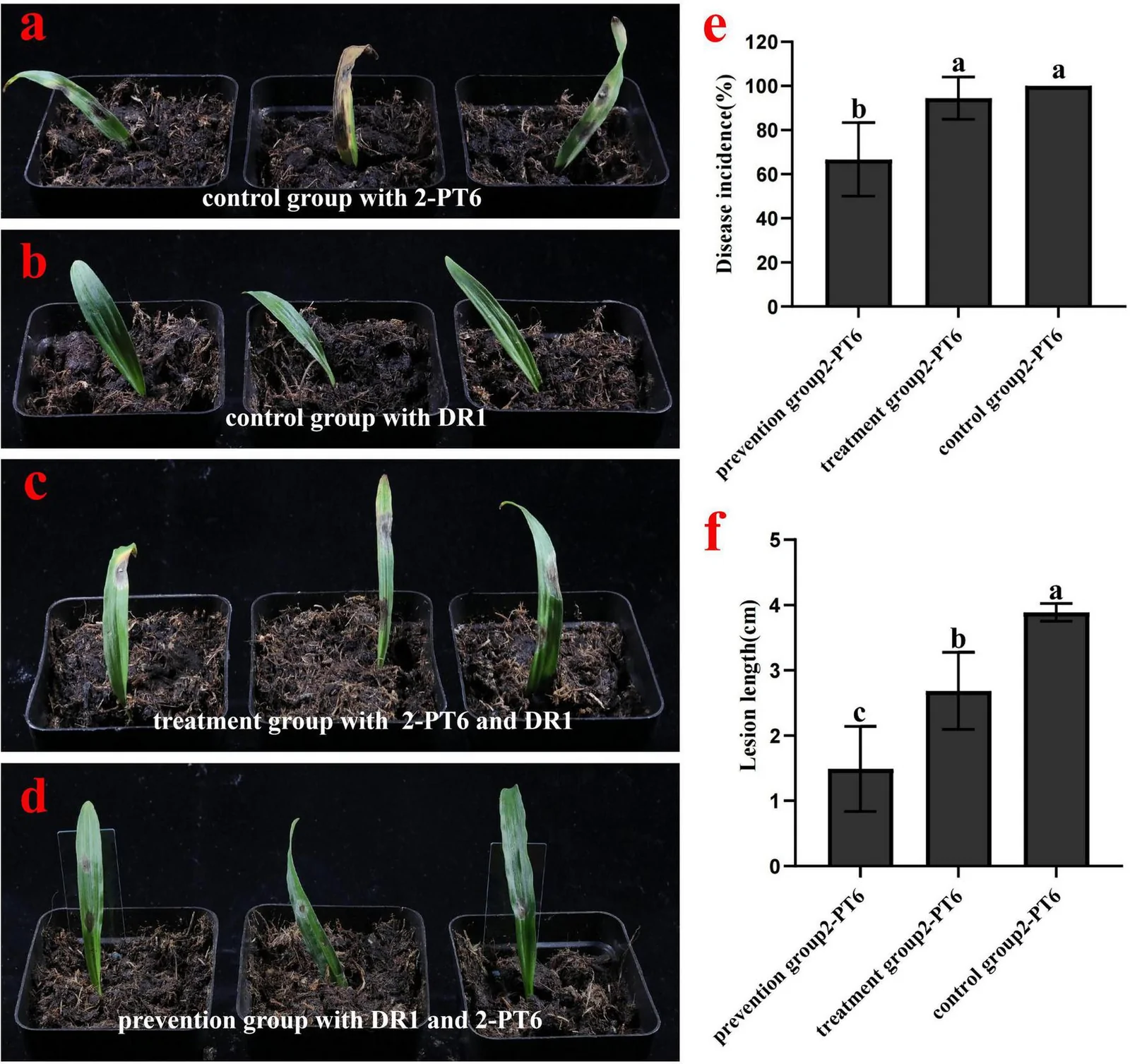
Control efficacy of *P. pergaminensis* DR1 against anthracnose on live *P. bulbocodioides* plants under indoor pot experiments at 12 dpi. **(a)** Positive control (inoculation with 2-PT6 alone); **(b)** Negative control (spraying with DR1 alone without pathogen); **(c)** Treatment (the same DR1 spray applied immediately after inoculation); **(d)** Prevention (DR1 suspension sprayed 24 h before inoculation with strain 2-PT6); **(e)** Disease incidence on leaves; **(f)** Lesion length on leaves. Data are presented as mean ± SD. Different lowercase letters above bars indicate significant differences among treatments based on one-way ANOVA followed by Duncan’s multiple range test (*P* < 0.05).

#### 3.3.2 Greenhouse experiment on natural disease

After 4 months of monthly spraying with DR1 suspension on naturally infested nursery beds (Figures 11a,b), significant disease control was achieved (Figures 11c,d), with boxplots visualizing data distributions (Figure 11e) and the text and Supplementary Table 4 reporting the means and statistical test results. The disease index in the treatment group (1.83 ± 2.91%) was significantly lower than that in the control group (10.64 ± 12.00%) (*P* < 0.05). Beta regression showed a significant treatment effect (*P* = 0.0088), and the estimated marginal means were used to calculate a field control efficacy of 62.5%.

**FIGURE 11:**
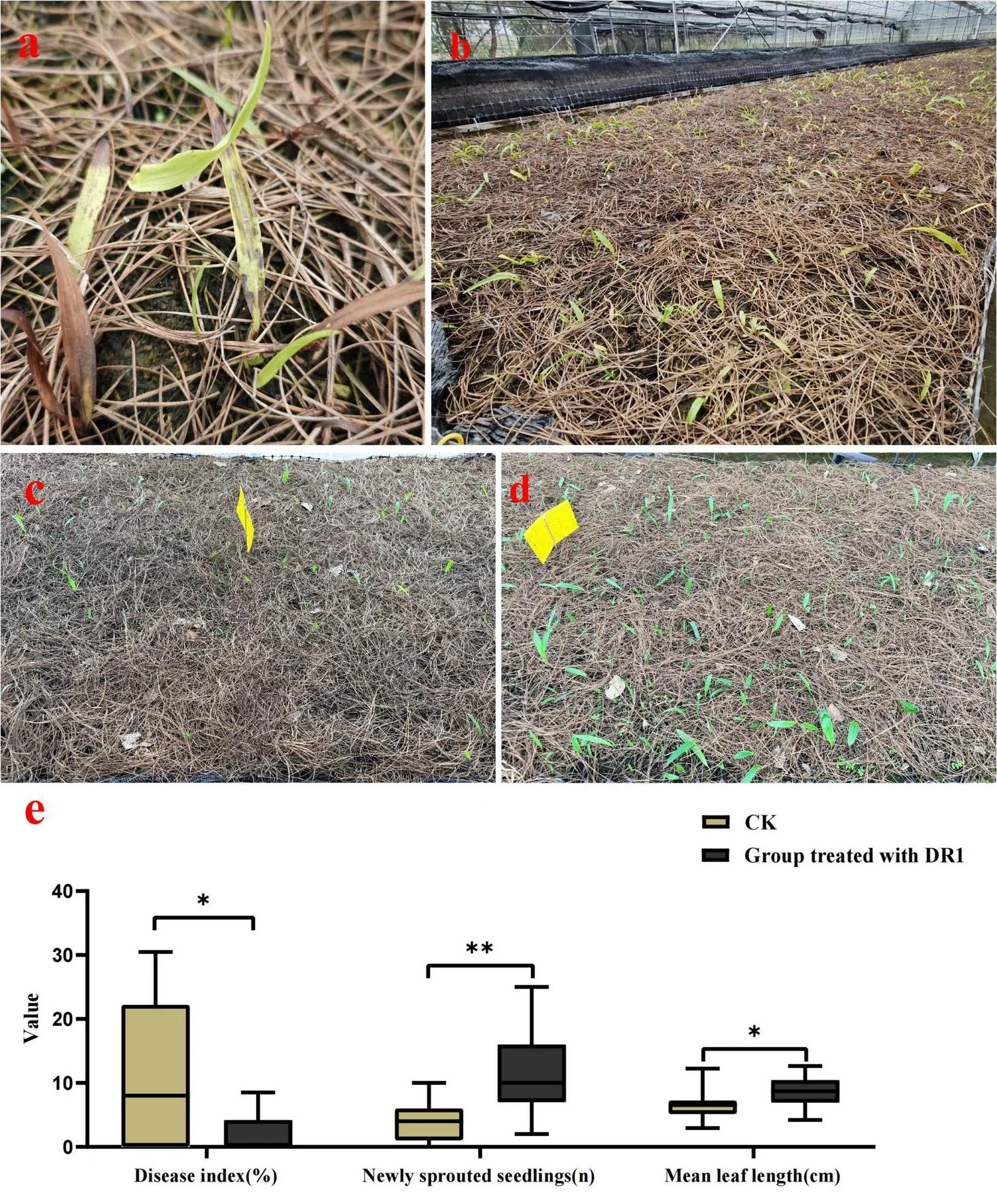
Disease control efficacy and growth promotion of *P. pergaminensis* DR1 on *P. bulbocodioides* after 4 months of continuous application in the greenhouse. **(a)** Close-up of a diseased sample before the experiment; **(b)** Diseased nursery beds before the experiment; **(c)** Control group (with five statistical plots) after four months of the experiment; **(d)** Nursery beds after four months of continuous application of DR1 suspension (with five statistical plots); **(e)** Boxplots comparing disease index, number of newly emerged leaves, and average leaf length between the treatment and control groups. Boxes represent the interquartile range (IQR), horizontal lines inside the boxes indicate medians, and whiskers extend to the minimum and maximum values within 1.5 × IQR. Although the boxplots display medians and quartiles, the significance asterisks (*, **) are derived from independent-samples *t*-tests comparing the means, and the corresponding mean ± SD values are reported in the Results section. Significance levels: **P < 0.01; *P < 0.05.

Plant growth was also promoted by DR1 treatment. A significantly higher number of newly emerged leaves per quadrat was observed in the treatment group (11.53 ± 6.36) than in the control group (3.87 ± 3.36) (*P* < 0.01), representing a 198% increase (2.98-fold) compared with the control. Likewise, a significantly greater average leaf length was found in the treatment group (8.64 ± 2.46 cm/leaf) than in the control group (6.60 ± 2.21 cm/leaf) (*P* < 0.05; independent samples *t*-test).

Collectively, these findings demonstrate that *P. pergaminensis* DR1 effectively controls anthracnose and promotes leaf growth in *P. bulbocodioides* under greenhouse conditions, corroborating its *in vitro* antagonistic activity and plant growth-promoting traits.

## 4 Discussion

In this study, two pathogenic strains, 2-PT5 and 2-PT6, were isolated from diseased *P. bulbocodioides* plants collected from a plantation in Qujing City, Yunnan Province. Based on morphological characteristics, multilocus sequence analysis (*ITS*, *CHS*, *TUB2*, *GAPDH*, and *ACT*), and phylogenetic reconstruction, both strains were identified as *C. orchidophilum*. This pathogen is a prevalent causal agent of orchid diseases and has been frequently reported in Yunnan Province in recent years. Yang et al. (2022) first reported *C. orchidophilum* causing anthracnose on *Bletilla striata*, with a disease incidence of up to 54.05%, highlighting the severe threat it poses to large-scale cultivation of medicinal orchids. Our findings are consistent with those reports and further confirm the widespread distribution of *C. orchidophilum* among orchids in Yunnan Province. Notably, although both strains clustered in the same clade as the reference strain CBS 632.80 and shared high sequence similarity, they exhibited significant phenotypic divergence in sporulation capacity. Under identical *in vitro* conditions, strain 2-PT6 produced up to 183-fold more conidia than strain 2-PT5, with the difference being statistically significant from day 5 onward (*P* < 0.0001). Bernasconi et al. (2022) pointed out that asexual reproductive potential (sporulation capacity) is a more reliable predictor of competitive ability among pathogen strains than virulence. Although this relationship was demonstrated in *Zymoseptoria tritici*, whether a similar pattern holds for *C. orchidophilum* requires further investigation. Future comparative genomic and pathogenicity assays are warranted to elucidate the molecular basis of this phenotypic divergence and its ecological implications for anthracnose management in *P. bulbocodioides* cultivation.

Biocontrol strategies for medicinal orchids are directly relevant not only to yield improvement but also to the safety and quality of medicinal materials, as chemical pesticide residues can compromise both safety and export value (Vaghela and Gohel, 2023; Wu et al., 2021). In recent years, endophytic bacteria from medicinal plants have gained increasing attention as biocontrol resources (Jiang et al., 2025; Zhu et al., 2025). In this study, a strain designated DR1 with strong antagonistic activity against *C. orchidophilum* was isolated from the roots of *P. bulbocodioides* and identified as *P. pergaminensis* based on multilocus phylogenetic analysis of 16S rRNA and the *gyrB*, *rpoB*, and *rpoD* genes. This study broadens the functional repertoire of *P. pergaminensis*. Beyond its plant-growth-promoting activity on wheat documented by Díaz et al. (2022), this species also exhibits potential for biocontrol applications.

During dual-culture assays, orange-red conidial masses were consistently observed at the inhibition zone margins of pathogen 2-PT6. Similar phenomena have been reported in the dual culture of *C. gloeosporioides* and *B. amyloliquefaciens*, and were associated with the upregulation of sporulation-related genes and activation of secondary metabolite pathways (Wang et al., 2020). DR1 exhibited detectable siderophore production and protease secretion activities, which may contribute to its antagonistic activity by chelating environmental iron ions and degrading fungal cell wall proteins (Köhl et al., 2019; Saha et al., 2016). The Oxford cup assay demonstrated that the cell-free extracellular filtrate of DR1 exerted no direct inhibitory effect on mycelial growth, indicating that DR1 does not produce stable, diffusible antifungal secondary metabolites under the conditions tested in this study (LB liquid medium). This result is consistent with the genomic information of the *P. pergaminensis* type strain 1008^T^, which lacks classical biocontrol gene clusters such as those for DAPG and pyoluteorin (Díaz et al., 2022). Furthermore, the VOC inhibition assay demonstrated that the volatile organic compounds produced by DR1 achieved inhibition rates of 53.04% and 48.08% against 2-PT5 and 2-PT6, respectively, confirming that volatile-mediated antagonism constitutes an important component of the biocontrol efficacy of DR1. The genome of the type strain *P. pergaminensis* 1008^T^ contains a *undA* homolog (Díaz et al., 2022), which in *Pseudomonas* spp. is responsible for the biosynthesis of the antifungal volatile 1-undecene (Rui et al., 2014). Notably, 1-undecene is a common component of VOCs produced by *Pseudomonas* spp., with documented antifungal and anti-oomycete activities. For instance, two olive-root-endophytic *Pseudomonas* strains, *Pseudomonas* sp. PICF6 and *P. simiae* PICF7, emit up to twenty volatile compounds including 1-undecene, several of which possess reported antifungal properties (Montes-Osuna et al., 2022). Pure 1-undecene has been shown to inhibit the oomycete *Phytophthora infestans* in a dose-dependent manner (Hunziker et al., 2015). Based on the close phylogenetic relationship between DR1 and the type strain 1008^T^, it is plausible that DR1 may possess a similar gene repertoire for volatile production, and 1-undecene may contribute to the VOC-mediated inhibition observed here. Nevertheless, direct volatile profiling via solid-phase microextraction coupled with gas chromatography–mass spectrometry (SPME-GC-MS) is still missing for DR1. Gene-knockout or heterologous-expression experiments are required to functionally validate the involvement of *undA* in DR1.

The indoor pot experiment revealed certain limitations in the mode of action of *P. pergaminensis* DR1. At 12 days post-inoculation, curative application of DR1 significantly restricted lesion expansion compared with the positive control (inoculated with the pathogen only), but did not significantly reduce disease incidence (*P* > 0.05). In contrast, preventive application significantly reduced both lesion length and disease incidence (*P* < 0.05). Notably, by 15 days post-inoculation, all diseased leaves in the curative treatment group had undergone complete rot, while those in the prevention group also exhibited total necrosis and rot by 18 days post-inoculation. A plausible explanation for this delayed but inevitable disease progression is the production of phytotoxins by *C. orchidophilum* once infection is established. *Colletotrichum* species are well-known producers of phytotoxins, including polyketides, terpenes, and alkaloids (Xu et al., 2021). Toxin-induced host necrosis has been demonstrated in several species: *C. dematium* produces phytotoxins that cause necrotic lesions on both host and non-host plants (Yoshida et al., 2000); colletochlorin A from *C. gloeosporioides* induces extensive leaf necrosis (Masi et al., 2018); and toxin metabolites of *C. lindemuthianum* and *C. truncatum* trigger necrotic lesions on legumes (Amusa et al., 1994). However, phytotoxin production by *C. orchidophilum* was not directly measured in this study and warrants further investigation. Consequently, although *P. pergaminensis* DR1 partially suppresses mycelial growth, phytotoxins, if produced by the pathogen during infection, may cause irreversible tissue damage that the biocontrol agent cannot reverse once infection has progressed beyond a certain stage. Nonetheless, the delay achieved by DR1 application, even if insufficient for curative eradication, can provide a critical window for integrated disease management under field conditions by enabling preventive application before substantial pathogen establishment. This interpretation was corroborated by our greenhouse trial: although monthly spraying of *P. pergaminensis* DR1 suspension was initiated when disease was already present and did not reverse the existing disease progression or halt the rotting of diseased leaves, after four months, the disease index, which was calculated based on the disease grades of all surveyed leaves rather than only newly emerged leaves, was significantly reduced, with a field control efficacy of 62.5%, and both the number of newly emerged leaves and leaf length were markedly increased. Therefore, based on the pot experiment and greenhouse trial in this study, we propose that to fully exploit the dual effects of *P. pergaminensis* DR1 in pathogen suppression and plant growth promotion under high disease pressure, preventive sprays should be implemented as early as possible, preferably at the early stage of disease susceptibility or right before disease outbreak, with repeated applications at 15- or 30-day intervals.

Although the genome of strain DR1 was not sequenced in the present study, the genome of the *P. pergaminensis* type strain 1008^T^ has been fully sequenced and systematically mined for plant-beneficial traits (Díaz et al., 2022). Given the close relatedness between DR1 and the type strain, it is plausible that they may share similar functional gene sets. Based on these genomic data, the potential mechanisms by which DR1 promotes plant growth can be tentatively inferred. Specifically, the genome of strain 1008^T^ harbors the *iaaMH* operon responsible for indole-3-acetic acid (IAA) biosynthesis via the indole-3-acetamide pathway (Spaepen et al., 2007; Duca et al., 2014). Genome mining identified genes potentially involved in the biosynthesis of plant-growth-stimulating volatiles 2,3-butanediol and acetoin (Ryu et al., 2003; Fincheira and Quiroz, 2018). It possesses the *pvd* gene cluster governing pyoverdine siderophore production (Vansuyt et al., 2007; Trapet et al., 2016). It also contains *gab* genes involved in γ−aminobutyric acid (GABA) uptake and catabolism, which may indirectly modulate plant stress-response processes (Díaz et al., 2022; Takeuchi, 2018). Nevertheless, these hypotheses await further experimental validation, including whole-genome sequencing of DR1 and targeted gene-inactivation assays. In addition, several endophytic bacteria have been reported to regulate secondary-metabolite accumulation in host plants (Alymanesh and Ghanbari, 2025; Guo et al., 2025). Whether *P. pergaminensis* DR1 can modulate the biosynthesis of pharmaceutically active components in *P. bulbocodioides* remains to be elucidated.

In addition to the biocontrol potential of *P. pergaminensis* DR1, we evaluated the *in vitro* toxicity of six chemical fungicides against *C. orchidophilum* to provide baseline data for future field efficacy comparisons and the development of integrated disease management strategies. Among the tested fungicides, 25% pyraclostrobin showed the strongest inhibitory activity, with EC_50_ values of 0.0060 mg/L against 2-PT6 and 0.0031 mg/L against 2-PT5, which is 1–5 orders of magnitude lower than those of the other fungicides-indicating its potential as a chemical option, pending field efficacy evaluation. These data will inform the selection of chemical controls in future field trials, enabling a systematic comparison between biocontrol and chemical strategies and facilitating the design of integrated management programs.

In summary, this study identified *C. orchidophilum* as the causal agent of anthracnose on *P. bulbocodioides*, and isolated a root endophytic bacterium, *P. pergaminensis* DR1, which exhibited potent antagonistic activity against the pathogen and multiple plant growth-promoting traits. Greenhouse trials demonstrated that preventive application of DR1 achieved a field control efficacy of 62.5% and significantly promoted leaf emergence and elongation, preliminarily validating its dual potential for disease control and growth promotion. Although the molecular mechanisms underlying these activities remain to be elucidated, this work provides a novel biocontrol resource for the green and sustainable cultivation of *P. bulbocodioides*. Future studies should focus on genome sequencing of DR1, multi-omics dissection of its antifungal and growth-promoting pathways, optimization of fermentation and formulation processes, and multi-site field evaluations with chemical controls to facilitate the integration of DR1 into practical disease management programs for medicinal orchids. In addition, future work will assess the effects of DR1 inoculation on the quantitative uptake of nitrogen, phosphorus, potassium, and other mineral nutrients in *P. bulbocodioides*, with the aim of providing direct physiological evidence for its biofertilization potential.

## Data Availability

The sequence data generated in this study have been deposited in NCBI GenBank, and the accession numbers are provided in the article. All other original contributions presented in the study are included in the article/Supplementary material; further inquiries can be directed to the corresponding authors.
