# Supplementary material for: Endophytic Pseudomonas pergaminensis DR1 controls anthracnose and promotes growth of medicinal orchid Pleione bulbocodioides
Source: Front Microbiol. 2026 Oct 12;17:1909299. doi: 10.3389/fmicb.2026.1909299 (PMC13696471; doi:10.3389/fmicb.2026.1909299)
Supplement: Supplementary file 1 [file Table_1.docx]

**Supplementary Tables**

**Supplementary Table S1 Primer sequences used for molecular identification in this study.**

| **Sequencing subject** | **Gene** | **Primer** | **Oligonucleotide sequence (5'-3')** | **Reference** |
| --- | --- | --- | --- | --- |
| ***Colletotrichum* sp.** | *ITS* | ITS1 | TCCGTAGGTGAACCTGCGG | White et al., 1990. |
|  |  | ITS4 | TCCTCCGCTTATTGATATGC |  |
|  | *ACT* | ACT-512 F | ATGTGCAAGGCCGGTTTCGC | Matos et al., 2025. |
|  |  | ACT-783R | TACGAGTCCTTCTGGCCCAT |  |
|  | *CHS* | CHS-79 F | TGGGGCAAGGATGCTTGGAAGAAG |  |
|  |  | CHS-354R | TGGAAGAACCATCTGTGAGAGTTG |  |
|  | *GAPDH* | GDF1 | GCCGTCAACGACCCCTTCATTGA |  |
|  |  | GDR1 | GGGTGGAGTCGTACTTGAGCATGT |  |
|  | *TUB2* | T1 | AACATGCGTGAGATTGTAAGT |  |
|  |  | Bt2b | ACCCTCAGTGTAGTGACCCTTGGC |  |
| ***Pseudomonas* sp.** | *rpoB* | LAPS | TGGCCGAGAACCAGTTCCGCGT | Ait Tayeb et al., 2005 |
|  |  | LAPS27 | CGGCTTCGTCCAGCTTGTTCAG |  |
|  | *rpoD* | PsEG30F | ATYGAAATCGCCAARCG | Mulet et al., 2009. |
|  |  | PsEG790R | CGGTTGATKTCCTTGA |  |
|  | *gyrB* | UP1 | GAAGTCATCATGACCGTTCTGCAYGCNGGNGGNAARTTYGA | Yamamoto and Harayama. 1995 |
|  |  | UP2r | AGCAGGATACGGATGTGCGAGCCRTCNACRTCNGCRTCNGTCAT |  |
|  | 16S rRNA | 27F | AGAGTTTGATCCTGGCTCAG | Weisburg et al., 1991 |
|  |  | 1492R | GGTTACCTTGTTACGACTT |  |

**Supplementary Table S2 Detection of plant growth-promoting and antagonistic traits of strain DR1**

| **Test** | **Medium Composition** | **Positive Indicator** | **Reference** |
| --- | --- | --- | --- |
| Siderophore production | CAS 60.5 mg, HDTMA 72.9 mg, FeCl₃·6H₂O 2.65 mg, NaH₂PO₄·2H₂O 295.3 mg, Na₂HPO₄·12H₂O 1.21 g, NH₄Cl 125 mg, KH₂PO₄ 37.5 mg, NaCl 62.5 mg, agar 15.0 g, distilled water 1000 mL, pH 6.8 | Orange-yellow halo around colony | Zhou et al., 2022 |
| Protease activity | Glucose 15.0 g, skim milk powder 20.0 g, KCl 0.5 g, MgSO₄·7H₂O 0.5 g, agar 15.0 g, distilled water 1000 mL | Clear hydrolysis zone around colony | Zhou et al., 2022 |

**Supplementary Table S3 Fungicides used in the study, their manufacturers, and pesticide registration numbers.**

| **Pesticide** | **Manufacturer** | **Pesticide Registration No.** **(China)** | **Test concentrations (mg/L)** |
| --- | --- | --- | --- |
| 25% pyraclostrobin SC | Hebei Chengyue Chemical Co., Ltd. | PD20161392 | 0.04, 0.125, 0.5, 2.0 |
| 100 g/L copper sulfate AS | Xilong Scientific Co., Ltd. | N/A | 100, 500, 1000 |
| 250 g/L difenoconazole EC | Syngenta (Shanghai) Crop Protection Co., Ltd. | PD20080730 | 0.01, 0.05, 0.1, 0.125, 1.25, 12.5 |
| 450 g/L prochloraz EW | FMC (Suzhou) Plant Protection Co., Ltd. | PD20150992 | 0.05, 0.1, 0.225, 0.5, 2.25, 22.5 |
| 25 g/L fludioxonil SC | Syngenta (Nantong) Crop Protection Co., Ltd. | PD20150099 | 0.2, 1.0, 5.0, 25.0, 125.0 |
| 90% carbendazim WP | Shandong Zhongxin Kenong Biotechnology Co., Ltd. | PD20161209 | 0.1, 0.3, 1.0, 3.0, 10.0 |

Note: The number of concentration gradients varied depending on the inhibitory spectrum of each fungicide determined in preliminary experiments. For copper sulfate, three concentrations were considered sufficient to cover the effective range, as higher concentrations caused complete inhibition while lower concentrations showed negligible effects.

**Supplementary Table S4. Comparison of disease index, number of newly emerged leaves, and leaf length between control and treatment groups.**

| **Variable** | **Group** | **Mean ± SD (original)** | **SEM** | **t** | **df** | **P‑value (t‑test)** | **Beta‑regression estimated mean (%) (95% CI)** | **P‑value (Wald)** |
| --- | --- | --- | --- | --- | --- | --- | --- | --- |
| Disease index (%) | Control | 10.64 ± 12.00 | 3.10 | 2.764 | 28 | 0.010 | 24.99 (16.74-35.56) | 0.0088 |
|  | Treatment | 1.83 ± 2.91 | 0.75 |  |  |  | 9.36 (4.88-17.20) |  |
| Newly emerged leaves (n) | Control | 3.87 ± 3.36 | 0.87 | -4.131 | 28 | <0.001 | – | – |
|  | Treatment | 11.53 ± 6.36 | 1.64 |  |  |  | – |  |
| Leaf length (cm) | Control | 6.60 ± 2.21 | 0.57 | -2.399 | 28 | 0.023 | – | – |
|  | Treatment | 8.64 ± 2.46 | 0.64 |  |  |  | – |  |

Note: Data are shown as mean ± SD (with SEM in parentheses or in separate column). For disease index, beta‑regression estimated marginal means and 95% confidence intervals (CI) are provided; the Wald test P‑value for the treatment comparison is also shown. t‑test degrees of freedom = 28 for all variables.
